# Self-consciousness negatively mediates the positive association between internalized weight bias and weight status in cross-cultural survey and brain imaging study

**DOI:** 10.3389/fpsyt.2025.1703291

**Published:** 2025-11-03

**Authors:** Yuko Nakamura, Karin Hayashi, Norihide Maikusa

**Affiliations:** ^1^ Center for Evolutionary Cognitive Sciences, Graduate School of Art and Sciences, The University of Tokyo, Tokyo, Japan; ^2^ University of Tokyo Institute for Diversity and Adaptation of Human Mind (UTIDAHM), Tokyo, Japan; ^3^ Department of Psychiatry, Toho University Sakura Medical Center, Chiba, Japan

**Keywords:** weight bias internalization, self-consciousness, body mass index, subgenual anterior cingulate cortex, precuneus

## Abstract

**Introduction:**

Weight bias internalization (WBI), where individuals adopt negative stereotypes about excess weight, is linked to adverse health outcomes. Although prior research indicates associations between WBI, weight status, and psychological factors linked to self-consciousness, these relationships remain unclear. Thus, this study examined these associations and the relationship between brain characteristics and WBI or self-consciousness.

**Methods:**

An online survey was conducted in Japan (n = 1946), South Korea (n = 500), Germany (n = 598), and the United States (n = 580) to assess WBI, self-consciousness, and body mass index (BMI). In Japanese samples, associations between brain structural (n = 120) or functional (n = 30) characteristics and WBI or self-consciousness were explored.

**Results:**

Self-consciousness negatively mediated the influence of WBI on BMI, varying across countries. Gray matter volume in the precuneus correlated positively with self-consciousness, while the subgenual anterior cingulate cortex (sACC) response to food reward correlated positively with WBI. Functional connectivity between the precuneus and sACC was positively associated with self-consciousness.

**Conclusion:**

Self-consciousness may reduce the impact of WBI on BMI, and the precuneus could be related to this self-consciousness effect, providing further insight into the interactions between WBI and self-consciousness.

## 1 Introduction

The prevalence of obesity has increased worldwide (1), with global adult obesity more than doubling in the last 30 years. The rapid rise in the prevalence of obesity has led to more research on the adverse effects of weight bias and weight bias internalization (WBI) (2). Weight bias is defined as holding negative stereotypes about people with higher weights, such as being lazy, lacking willpower, or having poor eating habits, and the resulting negative attitudes toward these individuals (3). WBI is often result of witnessing or experiencing weight-based stigma (4), and occurs when individuals apply negative weight stereotypes to themselves and self-derogate because of their body weight (5). WBI tends to be more pronounced in women (6) and individuals with higher body weights (7). WBI is associated with poor psychosocial (e.g., negative mental health), physical (e.g., health-related quality of life), and behavioral (e.g., disordered eating) health (8).

The associations between WBI and body mass index (BMI) or weight change have been well studied (2). For instance, higher WBI is associated with greater BMI even in a predominantly healthy weight population (n = 1454) (9). Additionally, WBI is negatively associated with weight loss (10) and positively associated with weight gain (11). WBI is also associated with various psychological factors, such as poor body image (11), elevated body dissatisfaction (12), and psychological distress (13), all of which are related to self-consciousness (14–16). Self-consciousness is defined as the awareness of oneself, including one’s body, actions, and thoughts, and how others perceive them (17). The concept of self-consciousness encompasses both private and public dimensions. Private self-consciousness is defined as the awareness of one’s personal inner feelings, while public self-consciousness is the concern about how one is perceived by others (18). Thus, WBI, which involves the process of being aware of weight stigma, applying it to oneself, and devaluing one’s self-worth, is likely related to self-consciousness. In fact, self-consciousness is believed to be linked to stigma consciousness, which refers to a focus on one’s stereotyped status (19). Self-consciousness also influences weight management (20) and eating behavior (21), thereby potentially influencing the association between WBI and BMI. Moreover, given that WBI is reported in individuals across the weight spectrum (22), excess weight may not be its sole cause, as self-consciousness could also contribute to WBI. However, the associations among WBI, BMI, and self-consciousness remain unclear. Therefore, the current study aims to examine the effect of self-consciousness on the relationship between WBI and BMI.

The current study also examined cultural differences in the associations among WBI, BMI, and self-consciousness across four countries (Japan, South Korea, Germany, and the United States). Cultural differences in self-consciousness have long been studied (23, 24) and are related to eating habits. For example, the association between public self-consciousness and the intention to eat a healthy diet was modulated by cultural differences (25). Additionally, as the majority of WBI research has been conducted in the United States among women with higher BMI (2), undertaking research in diverse cultures could provide a better understanding of WBI.

Furthermore, the current study examined the associations between WBI or self-consciousness and the brain properties to investigate the neural mechanisms underlining WBI, considering the subgenual anterior cingulate cortex (sACC) as the region of interest (ROI). The sACC is a subregion of the cingulate cortex and has a wide-ranging neural connection with limbic, prefrontal, parietal, and mesiotemporal areas (26). The sACC contributes to the modulation of emotional behavior (27) and food-reward processing (28); self-awareness, encompassing the reflection of one’s recent behavioral history (29); and social decision-making, specifically the processing of prediction errors in social interactions (i.e., social prediction errors), such as the calculation of differences between actual and predicted intentions of others (30, 31). Thus, the sACC could play a significant role in self-consciousness by monitoring one’s thoughts and speculating on how others perceive them. Furthermore, given its involvement in observation-driven social learning (32), sACC could be associated with WBI by facilitating learning weight stigma and internalizing it. Together, the sACC is involved in social and personal interactions, which are significantly related to WBI and self-consciousness (5, 17). Additionally, the sACC plays a role in ingestive behavior since it is involved in processing various stimulus values (33), such as money (34) and food (35, 36), as well as food reward anticipation (37). Therefore, using the sACC as the ROI, magnetic resonance imaging (MRI) measurements of brain structure and responses to food reward were conducted as preliminary research in a Japanese sample.

It was hypothesized that greater WBI would be linked to heightened self-consciousness, as self-consciousness focuses on one’s body and how others see it (17). Since self-consciousness is involved in maladaptive eating (21), it could positively mediate the association between WBI and weight status. Due to cultural differences in self-consciousness (23, 24), its mediating effect would differ across countries. Furthermore, the sACC could contribute to increasing WBI by playing a role in self-awareness (29) and predicting the intentions of others (30, 31).

## 2 Materials and methods

### 2.1 Experimental design

First, an online survey was administered to assess WBI, self-consciousness, and BMI in Japan, South Korea, Germany, and the United States. Then, structural and functional MRI (fMRI) measures were performed on Japanese young adults. All studies were approved by the Ethics Committee of the Department of Arts and Sciences, University of Tokyo (Approval No. 812-2). This study adhered to the principles of the Declaration of Helsinki.

### 2.2 Participants

#### 2.2.1 Online survey

Participants aged 18–45 who had lived in the same country for more than 10 years were included in the online survey. Commercial survey sampling and administration companies were contracted to recruit participants and administer the online survey. After excluding those who failed to enter their responses, a total of 1946 participants from Japan, 500 from South Korea, 598 from Germany, and 580 from the United States were included in the statistical analyses (**Tables 1**, **2**; **Supplementary Table S1**, **Supplementary Figure S1**, **Supplementary Information**). Before beginning the online survey, participants were asked to provide their consent to contribute their data for research purposes; those who consented to participate in this research were included. Participants provided their age, gender, weight, and height, and completed questionnaires about WBI and self-consciousness.

**Table 1:** Demographic characteristics of the participants in the Japanese sample.

	Total	Men	Women	Gender difference
	(n = 1946)	(n = 689)	(n = 1257)	P-value*
Age (years) Mean ± SD (Range)	35.38 ± 6.90 (18–45)	36.43 ± 6.79 (18–45)	34.81 ± 6.89 (18–45)	**< 0.001**
Body mass index (kg/m2) Mean ± SD (Range)	21.53 ± 3.62 (14.87–42.24)	22.72 ± 3.65 (14.87–42.24)	20.88 ± 3.43 (13.79–41.40)	**< 0.001**
Weight Self-Stigma Questionnaire				
Self-devaluation Mean ± SD (Range)	15.91 ± 6.35 (6–30)	14.66 ± 5.97 (6–30)	16.60 ± 6.44 (6–30)	**< 0.001**
Fear of enacted stigma Mean ± SD (Range)	14.23 ± 4.68 (6–30)	13.71 ± 4.29 (6–27)	14.52 ± 4.86 (6–30)	**0.002**
Self-Consciousness Scale				
Public self-consciousness Mean ± SD (Range)	51.12 ± 13.29 (11–77)	47.07 ± 13.51 (11–77)	53.34 ± 12.64 (11–77)	**< 0.001**
Private self-consciousness Mean ± SD (Range)	45.28 ± 10.79 (10–70)	43.51 ± 11.12 (10–70)	46.25 ± 10.48 (10–70)	**< 0.001**

*Wilcoxon rank-sum test. SD, Standard deviation.

The bolded values indicate statistical significance at p < 0.05.

**Table 2:** Demographic characteristics of the participants in the samples of three countries.

South Korea					
	Total (n = 500)	Men (n = 245)	Women (n = 255)	Other (n = 0)	Gender difference (men vs women) P-value*
Age (years) Mean ± SD (Range)	34.15 ± 7.48 (15–45)	35.01 ± 7.29 (18–45)	33.32 ± 7.58 (18–45)	–	**0.011**
Body mass index (kg/m2) Mean ± SD (Range)	22.92 ± 3.74 (14.78–41.89)	24.33 ± 3.42 (15.54–41.89)	21.56 ± 3.53 (14.78–36.13)	–	**< 0.001**
Race	Asian: 489 White/Caucasian: 7 Black/African American: 1 Prefer not to answer/Don't know: 3	Asian: 243 White/Caucasian: 1 Black/African American: 1 Prefer not to answer/Don't know: 0	Asian: 246 White/Caucasian: 6 Black/African American: 0 Prefer not to answer/Don't know: 3	–	–
Weight Self-Stigma Questionnaire					
Self-devaluation Mean ± SD (Range)	17.89 ± 5.05 (6–30)	17.56 ± 4.86 (6–30)	18.21 ± 5.22 (6–30)	–	0.239
Fear of enacted stigma Mean ± SD (Range)	15.14 ± 5.47 (6–30)	14.97 ± 5.35 (6–30)	15.30 ± 5.58 (6–30)	–	0.584
Self-Consciousness Scale					
Public self-consciousness Mean ± SD (Range)	24.85 ± 5.20 (7–35)	24.66 ± 5.35 (7–35)	25.04 ± 5.06 (9–35)	–	0.388
Private self-consciousness Mean ± SD (Range)	34.35 ± 5.84 (10–50)	34.51 ± 5.77 (10–50)	34.20 ± 5.92 (16–48)	–	0.375
Germany					
	Total (n = 598)	Men (n = 284)	Women (n = 249)	Other ( n = 4)	P-value*
Age (years) Mean ± SD (Range)	30.37 ± 8.08 (18–45)	31.74 ± 7.19 (18–45)	29.16± 8.05 (18–45)	26.25 ± 8.10 (20–38)	**< 0.001**
Body mass index (kg/m2) Mean ± SD (Range)	24.79 ± 5.39 (15.00–53.33)	25.42 ± 5.33 (16.07–53.33)	24.20 ± 5.33 (15.00–52.47)	26.60 ± 9.81 (18.94–40.75)	**< 0.001**
Race	Asian: 22 White/Caucasian: 517 Black/African American: 17 Hispanic/Latino: 11 Native American: 1 Prefer not to answer/Don't know: 30	Asian: 5 White/Caucasian: 257 Black/African American: 9 Hispanic/Latino: 4 Native American:0 Prefer not to answer/Don't know: 9	Asian: 17 White/Caucasian: 259 Black/African American: 8 Hispanic/Latino: 6 Native American:1 Prefer not to answer/Don't know: 19	Asian: 0 White/Caucasian: 1 Black/African American: 0 Hispanic/Latino: 1 Native American:0 Prefer not to answer/Don't know: 2	–
Weight Self-Stigma Questionnaire					
Self-devaluation Mean ± SD (Range)	15.43 ± 6.18 (6–30)	15.99 ± 5.77 (6–30)	14.98 ± 6.50 (6–30)	11.50 ± 6.81 (6–20)	**0.031**
Fear of enacted stigma Mean ± SD (Range)	14.24 ± 6.18 (6–30)	14.87 ± 6.07 (6–30)	13.68± 6.24 (6–30)	13.50 ± 7.55 (6–20)	**0.010**
Self-Consciousness Scale					
Public self-consciousness Mean ± SD (Range)	22.46 ± 5.72 (7–35)	21.99 ± 5.43 (7–35)	22.90± 5.90 (7–35)	22.25 ± 10.37 (7–30)	0.077
Private self-consciousness Mean ± SD (Range)	32.11 ± 6.16 (16–50)	32.13 ± 6.03 (16–47)	15.50 ± 5.21 (17–50)	30.50± 9.54 (18–41)	0.989
United States					
	Total (n = 580)	Men (n = 282)	Women (n = 296)	Other ( n = 2)	P-value*
Age (years) Mean ± SD (Range)	33.79± 7.19 (18–45)	33.33 ± 7.18 (18–45)	32.11 ± 6.16 (16–50)	23.00 ± 5.66 (19–27)	0.089
Body mass index (kg/m2) Mean ± SD (Range)	27.24± 6.98 (12.37–58.27)	26.38 ± 6.32 (12.37–58.24)	28.10 ± 7.47 (16.12–58.27)	20.01± 0.31 (19.79–20.23)	**0.008**
Race	Asian: 25 White/Caucasian: 314 Black/African American: 164 Hispanic/Latino: 59 Native American:12 Prefer not to answer/Don't know: 6	Asian: 17 White/Caucasian: 139 Black/African American: 84 Hispanic/Latino: 32 Native American: 7 Prefer not to answer/Don't know: 3	Asian: 7 White/Caucasian: 175 Black/African American: 79 Hispanic/Latino: 27 Native American:5 Prefer not to answer/Don't know: 3	Asian: 1 White/Caucasian: 0 Black/African American: 1 Hispanic/Latino: 0 Native American:0 Prefer not to answer/Don't know: 0	
Weight Self-Stigma Questionnaire					
Self-devaluation Mean ± SD (Range)	15.63± 6.32 (6–30)	14.99 ± 6.01 (6–30)	16.23 ± 6.58 (6–30)	15.50 ± 3.54 (13–18)	**0.021**
Fear of enacted stigma Mean ± SD (Range)	15.63± 6.59 (6–30)	15.12 ± 6.49 (6–30)	16.14 ± 6.67 (6–30)	11.50 ± 3.54 (9–14)	0.079
Self-Consciousness Scale					
Public self-consciousness Mean ± SD (Range)	22.80 ± 6.23 (7–35)	22.26 ± 6.36 (7–35)	23.31± 6.08 (7–35)	24.50 ± 2.12 (23–26)	0.073
Private self-consciousness Mean ± SD (Range)	33.53 ± 6.22 (18–49)	33.41 ± 6.49 (18–49)	33.62 ± 5.97 (18–49)	37.50 ± 4.95 (34–41)	0.622

*Wilcoxon rank-sum test.

SD, Standard deviation.

The bolded values indicate statistical significance at p < 0.05.

#### 2.2.2 Brain measurements

A total of 120 Japanese participants were included (**Table 3**). All participants provided written informed consent. Those with a history of neurological injury; known genetic or medical disorders; previous or current use of psychotropic medications, tobacco, cigarettes, electronic cigarettes; and any MRI exclusion criteria were excluded from the study. Of the participants included in the brain structural measurements, 30 underwent the fMRI experiment (**Table 3**).

**Table 3:** Demographic characteristics of the participants who completed brain measurements.

Structural MRI measurement				
	Total (n = 120)	Men (n = 60)	Women (n = 60)	Gender difference (men *vs* women) P-value*
Age (years) Mean ± SD (Range)	24.88 ± 5.65 (20–44)	24.77 ± 5.54 (20–40)	25.00 ± 5.81 (20–44)	0.661
Body mass index (kg/m2) Mean ± SD (Range)	21.27± 2.81 (16.85–30.96)	21.09 ± 2.79 (16.94–30.96)	21.45 ± 2.83 (16.85–28.32)	0.442
Weight Self-Stigma Questionnaire				
Self-devaluation Mean ± SD (Range)	13.99 ± 6.10 (6–27)	17.03 ± 5.22 (7–27)	10.95 ± 5.39 (6–27)	**< 0.001**
Fear of enacted stigma Mean ± SD (Range)	11.15 ± 3.96 (6–25)	12.90 ± 4.29 (6–25)	9.40 ± 2.64 (6–16)	**< 0.001**
Self-Consciousness Scale				
Public self-consciousness Mean ± SD (Range)	54.46± 10.46 (27–74)	55.82 ± 11.28 (28–74)	53.10 ± 9.47 (27–70)	0.065
Private self-consciousness Mean ± SD (Range)	50.75± 9.27 (21–70)	50.40 ± 9.38 (25–70)	51.10 ± 9.23 (21–68)	0.592

Functional MRI measurement				
	Total	Men	Women	Gender difference (men *vs* women)
	(n = 30)	(n = 20)	(n = 10)	P-value^$^
Age (years) Mean ± SD (Range)	24.77 ± 5.04 (20–36)	24.30 ± 4.40 (20–36)	25.70 ± 6.29 (20–35)	0.982*
Body mass index (kg/m2) Mean ± SD (Range)	21.26 ± 2.72 (16.85–28.32)	21.32 ± 3.09 (16.85–28.32)	21.13 ± 1.91 (18.85–25.07)	0.859
Weight Self-Stigma Questionnaire				
Self-devaluation Mean ± SD (Range)	12.47 ± 5.83 (6–27)	10.75 ± 5.32 (6–27)	15.90 ± 5.49 (7–25)	**0.020**
Fear of enacted stigma Mean ± SD (Range)	10.67 ± 2.90 (6–18)	10.00 ± 2.53 (6–16)	12.00 ± 3.27 (7–18)	0.075
Self-Consciousness Scale				
Public self-consciousness Mean ± SD (Range)	56.00 ± 9.54 (31–71)	56.70 ± 7.95 (39–68)	54.50 ± 12.50 (31–71)	0.561
Private self-consciousness Mean ± SD (Range)	52.03 ± 7.70 (40–68)	52.90 ± 8.08 (40–68)	50.30 ± 6.93 (41–62)	0.393

*Wilcoxon rank-sum test. ^$^=Tow-sample t-test. MRI, magnetic resonance imaging; SD, Standard deviation.

The bolded values indicate statistical significance at p < 0.05.

### 2.3 Measurements

#### 2.3.1 Measurements for WBI and self-consciousness

To assess WBI, the weight self-stigma questionnaire (WSSQ) (38) was used (**Supplementary Table S2**). The WSSQ comprises two subscales: self-devaluation (negative thoughts about being overweight) and fear of enacted stigma (the perception of being discriminated and identification with a stigmatized group). To assess public- and private self-consciousness, the self-consciousness scale (SCS) (18) was used (**Supplementary Table S3**, **Supplementary Material**).

#### 2.3.2 Brain imaging

##### 2.3.2.1 Structural brain measurements

On the day of the MRI measurement, participants’ heights and weights were measured. Then, they were instructed to answer the WSSQ and SCS. Subsequently, they were escorted to the MRI scanner room.

All structural images of the brain were collected using a MAGNETOM Prisma 3.0 Tesla scanner with a 32-channel head coil (Siemens Healthineers, Erlangen, Germany) with a T1-weighted 3D MPRAGE protocol (repetition time (TR) = 1900 ms, echo time (TE) = 2.53 ms, flip angle = 9°, field-of-view (FOV) = 256 × 256 mm^2^, resolution 1.0 × 1.0 × 1.0 mm^3^).

##### 2.3.2.2 Functional brain measurements

###### 2.3.2.2.1 Experimental procedures for functional brain measurements

**Figure 1:**
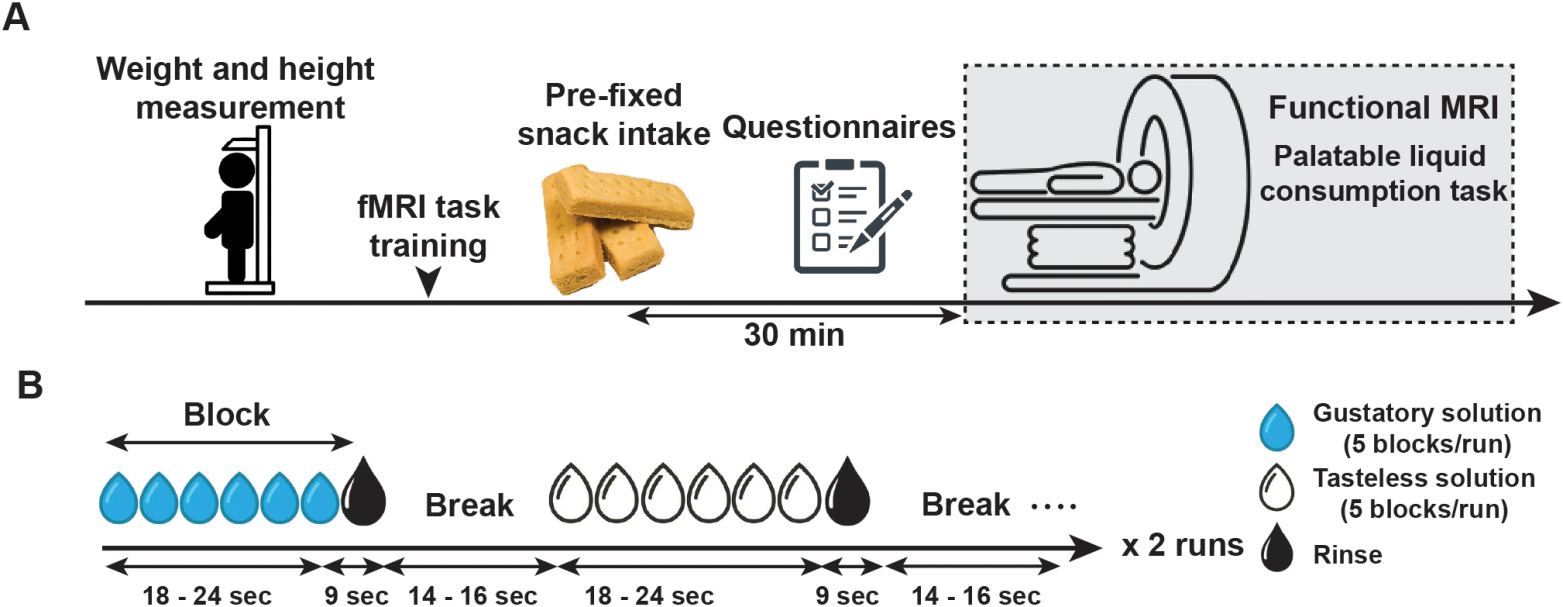
Outline of the experimental procedure and palatable liquid consumption task. **(A)** Experimental procedure. On the day of the fMRI experiment, participants were instructed to fast for at least 3 h before their visit. After they arrived at our laboratory, their weight and height were measured. Following anthropometric measurements, short training sessions for fMRI scans were conducted. Participants were then instructed to consume a pre-fixed snack (300 kcal) to standardize their internal state of hunger and fullness 30 minutes before the fMRI scan. Then, they completed the self-conscious scale and the weight self-stigma questionnaire. Subsequently, they were escorted to the MRI room. In the scanner, participants were instructed to rate their hunger and fullness. Then, they tasted a small amount of gustatory solution and rated liking, wanting, intensity, and familiarity of the solution. **(B)** Palatable liquid consumption task. During this task, a yogurt-flavored gustatory solution and the tasteless solution were delivered randomly in five blocks for each solution within a run. Within a block, a solution was delivered six to eight times (18–24 s) following three rinses (9 s) with a tasteless solution. The next block began after a short break (14–16 s). All participants underwent two runs of fMRI scans.

All participants underwent fMRI while consuming a gustatory stimulus (**Figure 1**). On the day of the fMRI experiment, participants were instructed to fast for at least 3 h before their visit. After they arrived at our laboratory, their weight and height were measured. Following anthropometric measurements, short training sessions for fMRI scans were conducted. Participants were then instructed to consume a pre-fixed snack (300 kcal) to standardize their internal state of hunger and fullness 30 minutes before the fMRI scan. Then, they completed the Japanese versions of the SCS and WSSQ. Subsequently, they were escorted to the MRI room and placed on the MRI scanner table. Before the fMRI scans, they rated their internal state of hunger and fullness on an 8-point Likert-type scale (1 = not at all and 8 = more than ever). Additionally, they rated liking (“How much do you like this juice?”), wanting (“How much do you want to drink this juice?”), intensity (“How strong is the taste of this juice?”), and familiarity (“How familiar are you with this juice?”) after consuming a small amount of the gustatory solution (**Supplementary Table S4**). The fMRI scans were conducted over two runs.

###### 2.3.2.2.2 Solutions

A commercially available yogurt-flavored soft drink was used as the gustatory solution (Calpis Co., Ltd., Tokyo, Japan). The tasteless solution consisted of major ions in saliva. First, four tasteless solutions were prepared, including the original (25 mM KCl + 2.5 mM NaHCO_3_) and solutions with 25%, 50%, and 75% of the original concentration. The participant tasted a small amount of each solution and selected the one that tasted “the most like nothing” as their tasteless solution.

###### 2.3.2.2.3 Delivery of solutions

Details of the solution delivery system are provided elsewhere (39). In brief, each solution was administered via a transparent polyvinyl chloride plastic tube affixed to the participant’s mouthpiece using electronic syringe pumps, which regulated the timing and volume of each solution. One delivery consists of 0.80 milliliters of the solution over a period of 2 s following a 1 s break.

###### 2.3.2.2.4 Functional MRI experiment

In the MR scanner, the participants performed two runs of the palatable liquid consumption task (**Figure 1**). This task involved the delivery of gustatory and tasteless solutions. Each solution was delivered randomly in five blocks for each run. Within a block, a solution was delivered six to eight times (18–24 s) following three rinses (9 s) with a tasteless solution. The next block began after a short break (14–16 s). During this task, a white fixation point on a black background was presented to participants through a mirror in front of them. They were instructed to gaze at the fixation point during the task. The average length of each run was 7 min 38 s ± 1.32 s (mean ± standard deviation (SD)).

###### 2.3.2.2.5 Functional imaging acquisition

All images were collected using a MAGNETOM Prisma 3.0 Tesla scanner with a 32-channel head coil (Siemens Healthineers, Erlangen, Germany). Anatomical images were acquired using the T1-weighted 3D MPRAGE protocol, which was also used for structural brain measurements, as mentioned above. As functional images, T2*-weighted images reflecting blood-oxygen-level-dependent (BOLD) signals were acquired using 2D gradient-echo echo-planar imaging (EPI) with an isotropic resolution (3.0 × 3.0 × 3.0 mm^3^), parallel acquisition factor = 3, TR = 2000 ms, TE = 25 ms, 39 slices, and flip angle = 80° in a 192 mm^2^ FOV, transverse slices with phase encoding in the P > > A direction. All the images were acquired in an interleaved manner.

### 2.4 Statistics

#### 2.4.1 Online survey

**Figure 2:**
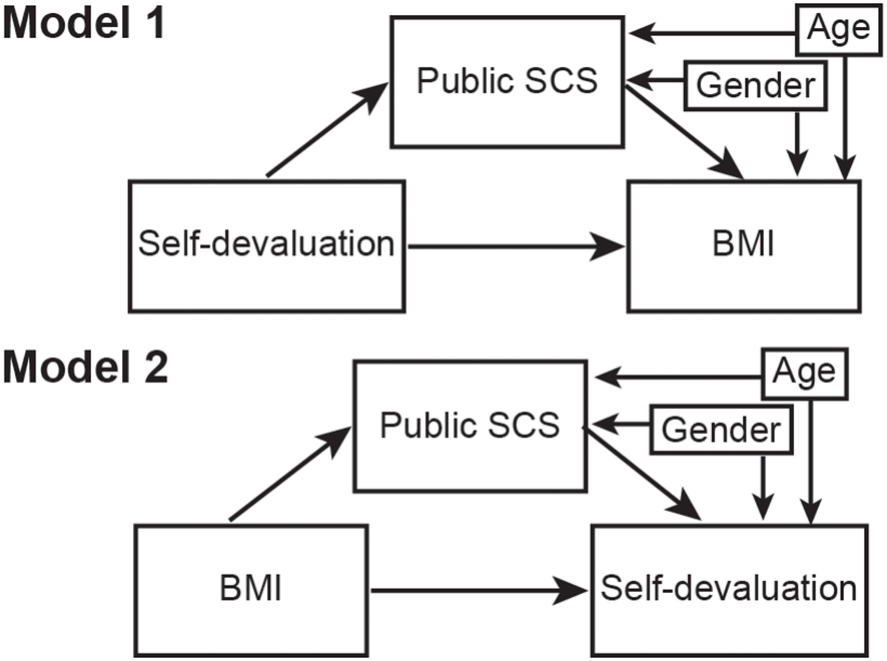
Mediation diagram indicating relationships across self-devaluation, body mass index (BMI), public self-consciousness of the Self-Consciousness Scale (public SCS), and gender. In Model 1, self-devaluation was set as a predictor of public SCS, public SCS was set as a predictor of BMI, and self-devaluation was set as a predictor of BMI. The mediation effect of public SCS on the path from self-devaluation to BMI was also estimated. In Model 2, BMI was set as a predictor of public SCS, public SCS was set as a predictor of self-devaluation, and BMI was set as a predictor of self-devaluation. The mediation effect of public SCS on the path from BMI to self-devaluation was also estimated. BMI: body mass index; Public SCS: public self-consciousness of the Self-Conscious Scale.

First, in Japanese samples, the gender differences in the WSSQ, SCS, age, and BMI were assessed (**Table 1**). Then, a multiple linear regression analysis was performed to test the associations between WBI, self-consciousness, and BMI. For this analysis, the model included BMI as the dependent variable and self-devaluation, fear of enacted stigma, public- and private SCS, age, and gender as the explanatory variables (see **Supplementary Information**). The same regression analysis was conducted separately for men and women, excluding gender. Based on the results of the regression analysis, a model comparison was performed to investigate the relationships between BMI, self-devaluation, and public SCS (**Figure 2**) using a cross-validation analysis. A cross-validation analysis was conducted with the cvsem package in R (40, 41) using k = 10 folds to evaluate the performance of two mediation models based on the Kullback-Leibler Divergence (KL-D). In Model 1, self-devaluation was set as a predictor of public SCS, public SCS was set as a predictor of BMI, and self-devaluation was set as a predictor of BMI. The mediation effect of public SCS on the path from self-devaluation to BMI was also estimated. In Model 2, BMI was set as a predictor of public SCS, and public SCS as well as BMI were set as predictors of self-devaluation. The mediation effect of public SCS on the path from BMI to self-devaluation was also estimated. In Model 1 and 2, the effects of gender and age were included as covariates to regress out their effects (**Figure 2**).

Next, the mediation effect of public SCS on the association between BMI and self-devaluation was examined using the best model suggested by the model comparison. A mediation analysis using the lavaan package in R (42) with 5,000 bootstrapped samples was conducted to assess the mediation effect of self-devaluation on BMI through public SCS, controlling for the effect of gender and age. For this analysis, all numeric variables were centered.

In South Korea, Germany, and the United States, the gender differences in the WSSQ, SCS, age, and BMI were assessed (**Table 2**). Then, the same regression models used in Japanese samples were fitted for data from each country. Subsequently, multi-group structural equation modeling (SEM) examined whether the mediation effect of public SCS differed in the four countries. Multi-group SEM was conducted using the lavaan package in R (42). For this analysis, numeric variables, except public SCS, were centered in each country. The Japanese version of the SCS was rated using a 7-point Likert scale, although the Korean, German, and original SCS were rated using a 5-point Likert scale. Thus, public SCS was centered and divided by the SD in each country to normalize data. First, in each country, a simple mediation model, including gender and age as covariates to control their effects, was estimated. Then, an omnibus Wald χ² test was conducted to assess equality of the indirect effect across the four countries, based on a covariance matrix of estimates from a nonparametric bootstrap (5,000 resamples). The same multi-group SEM was performed in men and women separately, without controlling for the effect of gender.

Additionally, the correlation coefficients between BMI and self-devaluation and those between BMI and public SCS were compared between Japan and the other countries. To compare the strength of correlations between Japan and other countries, Fisher’s z-transformation was applied to the correlation coefficients, and a two-tailed z-test was performed for independent correlations. The statistical significance threshold for this comparison was set at p < 0.016 (0.05/3) using the Bonferroni adjustment.

#### 2.4.2 Brain imaging measurement

For structural images, voxel-based morphometry (VBM) was applied. Conventional preprocessing, including skull-strapping, segmentation, normalization, and smoothing, was performed. Subsequently, a voxel-wise general linear model (GLM) including preprocessed grey matter images as a dependent variable, self-devaluation or public SCS as explanatory variables, and BMI and estimated total intracranial volume as nuisance variables was applied. The predicted effect of these analyses was tested using an ROI approach. For the ROI, a sphere with a radius of 10 mm centered on [x, y, z] = [-1, 27, -2.3] in the sACC was set based on previous studies (29, 31). The unpredicted effects were tested using whole brain analysis (**Supplementary Material**).

On functional images, conventional preprocessing was performed using Statistical Parametric Mapping 12 (SPM12) software (43) with the CONN functional connectivity toolbox (CONN, version 22a) (44, 45), including slice-timing correction, field map correction, realigning and unwarping, normalization onto the standard Montreal Neurological Institute space, and smoothing. Detailed preprocessing procedures are provided in the **Supplementary Material**.

Preprocessed functional images were brought to the individual level (first-level). The condition-specific effects (gustatory, tasteless, and rinse) at each voxel were estimated using a GLM. The canonical hemodynamic response function provided by SPM12 was used to model the responses to the events. In the time-series analysis, a high-pass filter (270 s) was included in the filtering matrix to remove low-frequency noise and slow drifts from the signal. Confounding regressors from the preprocessing stage were also included in the model as covariates of no interest. Then, the [gustatory > tasteless] contrast image was created for individual participants.

The [gustatory > tasteless] contrast image was used in the subsequent group-level (second-level) analysis. To assess the correlations between the individual [gustatory > tasteless] contrast image and self-devaluation or public SCS, a group-level voxel-wise linear regression analysis with self-devaluation or public SCS as a covariate-of-interest and gender and age as covariate-of-no-interests was performed on the individual [gustatory > tasteless] contrast images. The predicted effect of these analyses was tested using the ROI approach. The unpredicted effect of these analyses was tested using whole brain analysis. For these analyses, voxelwise thresholding with the family wise error rate (FWE) correction based on random field theory implemented in SPM12 (46) was applied. The threshold was set at p_FWE-corrected_ < 0.05.

Then, for seed to whole brain functional connectivity analysis, generalized psychophysiological interaction (gPPI) (47, 48) analysis was adapted using preprocessed functional images. For this, a sphere with a radius of 10 mm centered on the peak voxel ([x, y, z] = [8, 32, -6]) of the results from the aforementioned fMRI analysis was used as a seed. At the individual level, separately, for each pair of seed and target areas, a gPPI was defined with BOLD time-series extracted from the seed as physiological factors, boxcar signals characterizing each individual gustatory or taste condition convolved with an SPM canonical hemodynamic response function as psychological factors, and the product of the two as psychophysiological interaction terms. Functional connectivity changes across conditions were characterized by the multivariate regression coefficient of the psychophysiological interaction terms in each model. At the group-level, for each individual voxel in each condition, a separate GLM was estimated, with first-level connectivity measures at this voxel as dependent variables and self-devaluation or public SCS as independent variables, and BMI was a covariate-of-no-interest. Subsequently, between conditions (gustatory and tasteless), differences in associations between connectivity measures and self-devaluation or public SCS were evaluated using multivariate parametric statistics. Inferences were drawn at the level of individual clusters. Cluster-level inferences were based on parametric statistics from Gaussian Random Field theory (49). Results were thresholded using a combination of a cluster-forming p < 0.001 voxel-level threshold, and a p_FWE-corrected_ < 0.05 cluster-size threshold (46).

Based on the results of the online survey and brain imaging in Japanese, self-devaluation may indirectly affect BMI through public self-consciousness, and this mediating effect of public self-consciousness would be influenced by the level of gray matter volumes in the precuneus. To test this hypothesis, a moderated mediation analysis, in which self-devaluation (X) predicts BMI (Y) indirectly via public self-consciousness (M), with the M→Y path moderated by gray matter volumes in the precuneus (W), was performed using the lavaan (42) and manymome (50) packages in R. Age and gender were included as covariates in this analysis. For this analysis, the moderator (W) was centered. The moderated mediation model was fit with maximum likelihood. To obtain robust inference for indirect and moderated effects, nonparametric bootstrapping (5,000 resamples) was used. Conditional indirect effects at the moderator’s mean ±1 SD were probed.

## 3 Results

### 3.1 Associations among WBI, BMI, and self-consciousness

BMI was positively associated with self-devaluation (β = 0.53, p < 0.001) and age (β = 0.04, p= 0.048), and negatively associated with public SCS (β = -0.12, p < 0.001) and gender (β = -0.60, p < 0.001) (**Supplementary Table S5**). In men, BMI was positively associated with self-devaluation (β = 0.57, p < 0.001) and age (β = 0.07, p = 0.032), and negatively associated with public SCS (β = -0.12, p = 0.002) (**Supplementary Table S5**). In women, BMI was positively associated with self-devaluation (β = 0.53, p < 0.001) and negatively associated with public SCS (β = -0.12, p < 0.001) (**Supplementary Table S5**).

The model comparison showed that Model 1 demonstrated a significantly lower average KL-D of 0.09 (Standard Error (SE) = 0.03), indicating that it better approximated the true distribution of the data than did Model 2, which had a KL-D of 3.06 (SE = 0.31). This suggested that Model 1 was more effective in explaining the data.

**Figure 3:**
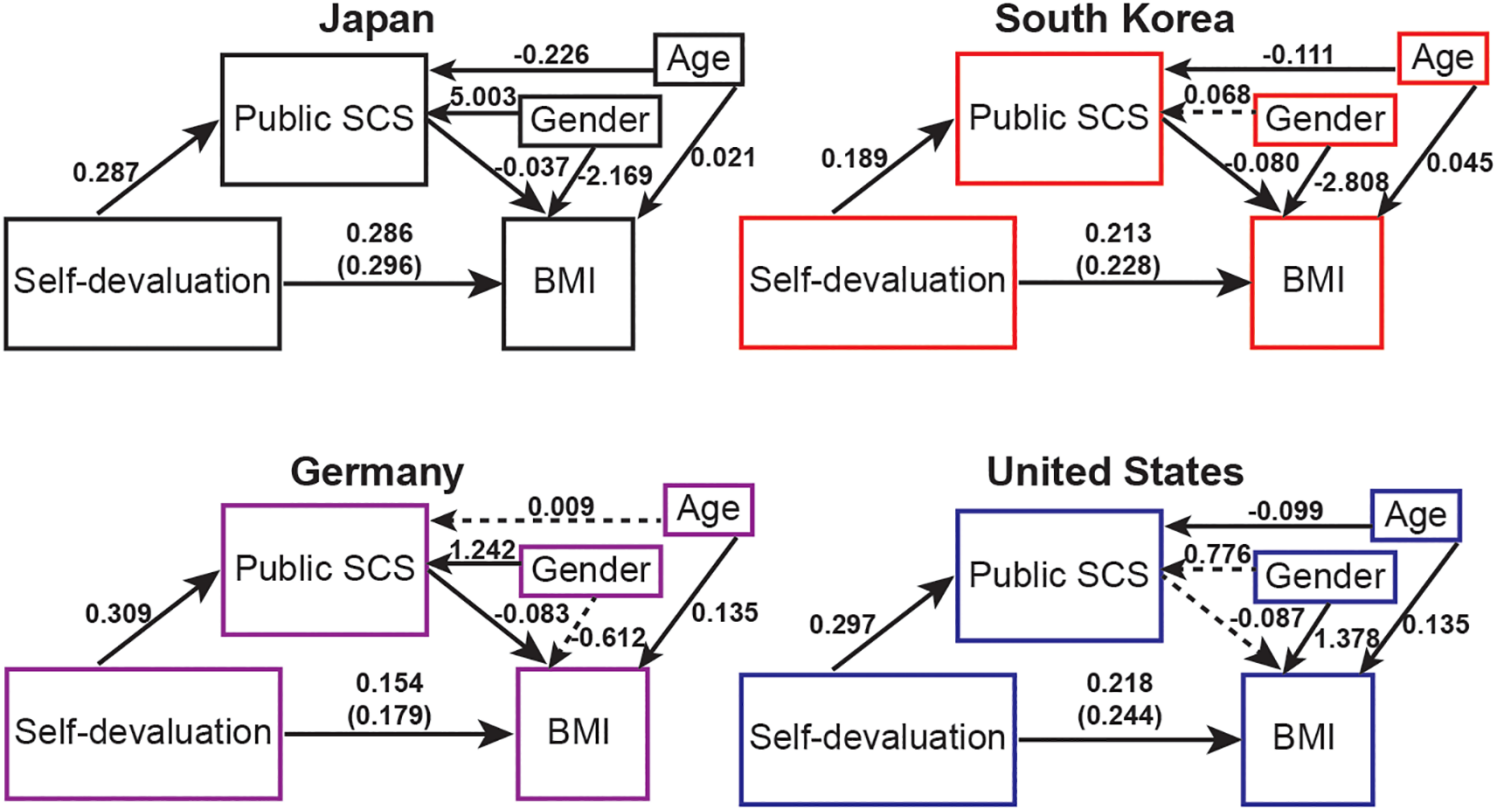
Associations among self-devaluation of the Weight Self-Stigma Questionnaire, public self-consciousness of the Self-Consciousness Scale (public SCS), and body mass index (BMI) in Japan, South Korea, Germany, and the United States. The standardized regression coefficients for the relationships are presented on the paths. The standardized regression coefficient between self-devaluation and BMI (direct effect), controlling for public self-consciousness and gender, is provided in parentheses. Dashed line paths indicate non-significance, p = 0.05 or more. Solid line paths indicate significance, p < 0.05. BMI: body mass index; Public SCS: public self-consciousness of the Self-Conscious Scale.

A significant indirect effect of self-devaluation on BMI through public SCS, with an estimate of -0.010 (SE = 0.003, 95% CI = [-0.016, -0.006], p < 0.001) (**Figure 3**) was observed. The overall model fit was adequate, with a comparative fit index (CFI) = 1.000, root mean square error of approximation (RMSEA) = 0.00, and standardized root mean square residual (SRMR) = 0.00, as values above 0.95 for CFI, below 0.08 for SRMR, and below 0.06 for RMSEA are acceptable (51). In men, the indirect effect of self-devaluation was not significant, with an estimate of -0.008 (SE = 0.004, 95% CI = [-0.017, -0.002], p = 0.056) demonstrating an overall adequate model fit, with a CFI = 1.000, RMSEA = 0.00, and SRMR = 0.00 (**Supplementary Figure S2**). In women, a significant indirect effect of self-devaluation was observed, with an estimate of -0.012 (SE = 0.003, 95% CI = [-0.019, -0.006], p < 0.001) demonstrating an overall adequate model fit, with a CFI = 1.000, RMSEA = 0.00, and SRMR = 0.00 (**Supplementary Figure S2**).

### 3.2 Cultural differences in associations among WBI, BMI, and self-consciousness

In South Korea, BMI was positively associated with self-devaluation (β = 0.31, p < 0.001) and age (β = 0.09, p = 0.037) and negatively associated with public SCS (β = -0.15, p = 0.008) and gender (β = -0.75, p < 0.001). In Germany, BMI was positively associated with self-devaluation (β = 0.23, p < 0.001) and age (β= 0.20, p < 0.001). In the United States, BMI was positively associated with self-devaluation (β = 0.23, p < 0.001), private SCS (β = 0.14, p = 0.009), age (β = 0.14, p < 0.001), and gender (β = 0.21, p = 0.011), and negatively associated with public SCS (β = -0.18, p = 0.001) (**Supplementary Table S5**). In men and women, GLM revealed varied associations among BMI, WBI, and self-consciousness. Detailed results can be found in **Supplementary Table S5**.

Although the mediation effects of public SCS differed across countries, these differences were not significant in total (χ² (3) = 2.74, p =0.43) (**Table 4**, **Figure 3**), in men (χ² (3) = 3.52, p = 0.32) (**Table 4**, **Supplementary Figure S2**), and in women (χ² (3) = 2.16, p = 0.54) (**Table 4**, **Supplementary Figure S2**).

**Table 4:** The standardized indirect effects of public self-consciousness on BMI through self-devaluation.

	β	Standard error	95% confidence interval (lower, upper)	z-value	p-value
Total (men and women)					
Japan	-0.010	0.003	-0.016, -0.006	-3.984	**< 0.001**
South Korea	-0.015	0.008	-0.034, -0.003	-1.922	0.055
Germany	-0.026	0.012	-0.051, -0.004	-2.188	**0.029**
United States	-0.026	0.015	-0.057, 0.001	-1.740	0.082
Men					
Japan	-0.008	0.004	-0.017, -0.002	-1.915	0.056
South Korea	-0.157	0.140	-0.485, 0.068	-1.119	0.263
Germany	-0.061	0.103	-0.272, 0.140	-0.589	0.556
United States	0.056	0.065	-0.054, 0.210	0.854	0.393
Women					
Japan	-0.012	0.003	-0.019, -0.006	-3.563	**< 0.001**
South Korea	-0.234	0.141	-0.564, -0.018	-1.664	0.096
Germany	-0.061	0.081	-0.260, 0.058	-0.754	0.451
United States	-0.072	0.088	-0.272, -0.082	-0.818	0.413

The bolded values indicate statistical significance at p < 0.05.

The correlation coefficient between BMI and self-devaluation, after adjusting for gender and age, was markedly higher in Japan (r = 0.54, p < 0.001) than in South Korea (r = 0.32, p < 0.001), Germany (r = 0.17, p < 0.001), and the United States (r = 0.20, p < 0.001). The differences in correlation coefficients were statistically significant: z = 5.49, p < 0.001 (South Korea *vs*. Japan); z = 9.08, p < 0.001 (Germany *vs*. Japan); z = 8.42, p < 0.001 (the United States *vs*. Japan) (**Supplementary Figure S3**). No significant correlations were observed between BMI and public SCS (ps > 0.13), and no significant differences in correlations were found between Japan and other countries (ps > 0.27). In men and women, the correlation coefficients between BMI and self-devaluation were significantly different in Japan than in other countries. In men, the correlation coefficient between BMI and self-devaluation was significantly greater in Japan (r = 0.55, p < 0.001) than in South Korea (r = 0.38, p < 0.001), Germany (r = 0.11, p = 0.071), and the United States (r = 0.07, p = 0.26). The differences in correlation coefficients were statistically significant: z = 3.01, p = 0.003 (South Korea *vs* Japan); z = 7.24, p < 0.001(Germany *vs* Japan); z = 7.78, p < 0.001 (the United States *vs* Japan) (**Supplementary Figure S3**). No significant correlations were observed between BMI and public SCS (ps > 0.24), and no significant differences in correlations were found between Japan and other countries (ps > 0.23). In women, the correlation coefficient between BMI and self-devaluation was significantly greater in Japan (r = 0.54, p < 0.001) than in South Korea (r = 0.30, p < 0.001), Germany (r = 0.23, p < 0.001), and the United States (r = 0.28, p < 0.001). The differences in correlation coefficients were statistically significant: z = 4.28, p < 0.001(South Korea *vs*. Japan); z = 5.95, p < 0.001 (Germany *vs* Japan); z = 4.86, p < 0.001(the United States *vs* Japan) (**Supplementary Figure S3**). No significant correlations were observed between BMI and public SCS (ps > 0.27), and no significant differences in correlations were found between Japan and other countries (ps > 0.59).

### 3.3 Associations among brain structural properties, WBI, and self-consciousness

No significant associations between self-devaluation or public SCS and gray matter volumes were observed in the predicted ROI.

**Figure 4:**
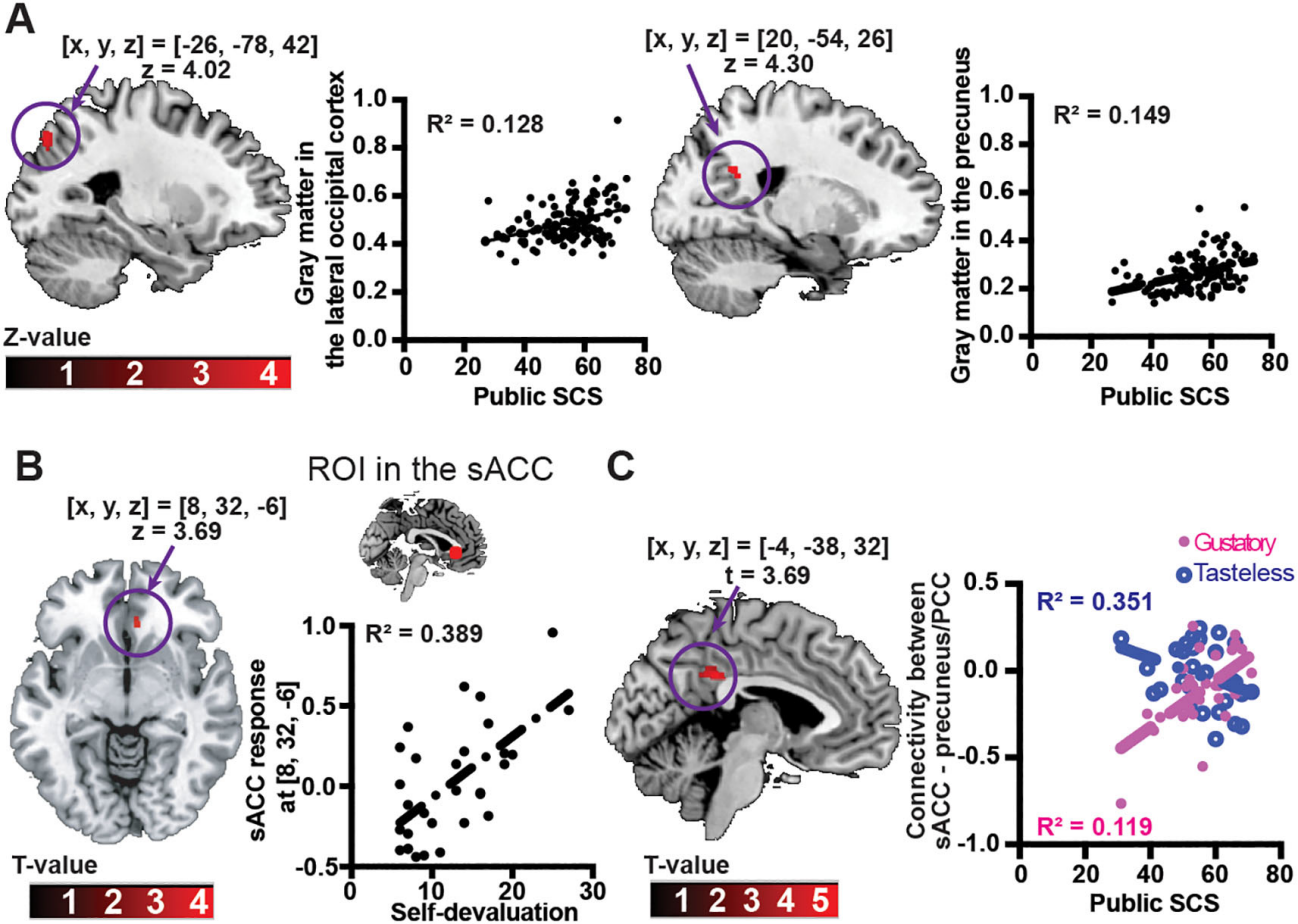
Associations between brain images and self-devaluation of the Weight Self-Stigma Questionnaire or public self-consciousness of the Self-Consciousness Scale. **(A)** Clusters with significant associations between public self-consciousness and gray matter volumes in the occipital cortex (left) and precuneus (right). The threshold was set at p_FWE-corrected_ < 0.05. Scatter plots indicate associations between gray matter volumes (y-axis) and public self-consciousness (x-axis). **(B)** A cluster with a significant association between self-devaluation and the subgenual anterior cingulate cortex (sACC) response to palatable liquid consumption. The threshold was set at p_FWE-corrected_ < 0.05. The scatter plot indicates the association between brain response (y-axis) and self-devaluation (x-axis). **(C)** A cluster showing a significant effect of condition on associations between connectivity with the sACC and public self-consciousness. The threshold was set at a combination of a cluster-forming p < 0.001 voxel-level threshold, and a p_FWE-corrected_ < 0.05 cluster-size threshold. The scatter plot indicates associations between sACC–precuneus/PCC connectivity (y-axis) and public self-consciousness (x-axis). R^2^ is the square of the correlation. Public SCS: public self-consciousness of the Self-Conscious Scale.

Whole brain analysis showed public SCS was positively associated with gray matter volumes in the lateral occipital cortex ([x, y, z] = [-26, -78, 42], z = 4.02, cluster size = 155 voxels, p_FWE-corrected_ = 0.036) and precuneus ([x, y, z] = [20, -54, 26], z = 4.30, cluster size = 16 voxels, p_FWE-corrected_ = 0.043) (**Figure 4a**). No association was found between gray matter volumes and self-devaluation. After adjusting for multiple comparisons due to the testing of gray matter volumes in relation to two variables, significance of associations between gray matter volumes in the lateral occipital cortex or precuneus and public SCS were at a trend level (p_FWE-corrected_ = 0.072 and 0.086, respectively).

### 3.4 Associations among brain response to palatable liquid consumption, WBI, and self-consciousness

Greater self-devaluation was positively associated with sACC response ([x, y, z] = [8, 32, -6], z = 3.69, p_FWE-corrected_ = 0.012, cluster size = 17 voxels) (**Figure 4b**), although public SCS did not reveal any significant association with sACC. After adjusting for multiple comparisons due to the testing of brain response in relation to two variables, the observed associations between sACC response and self-devaluation remained statistically significant (p_FWE-corrected_ = 0.024). Although whole brain analysis with a threshold of p_FWE-corrected_ < 0.05 showed no significant results, whole brain analysis with a softer threshold (p_uncorrected_ < 0.001 and cluster size > 20 voxels) exhibited an association between brain response and self-devaluation ([x, y, z] = [8, 32, -6], z = 3.69, p_uncorrected_ < 0.001, cluster size = 50 voxels), though not with public SCS.

The gPPI analysis showed that, in contrast to the tasteless condition, a significantly greater association was observed between the public SCS and the connectivity between the sACC and the cluster in the posterior cingulate cortex (PCC)/precuneus region ([x, y, z] = [-4, -38, 32], t = 3.69, p_FWE-corrected_ = 0.019, cluster size = 84 voxels) in the gustatory condition (**Figure 4c**). No association was found between connectivity with the sACC and self-devaluation.

### 3.5 Associations among WBI, self-consciousness, gray matter volumes in the precuneus, and BMI

**Figure 5:**
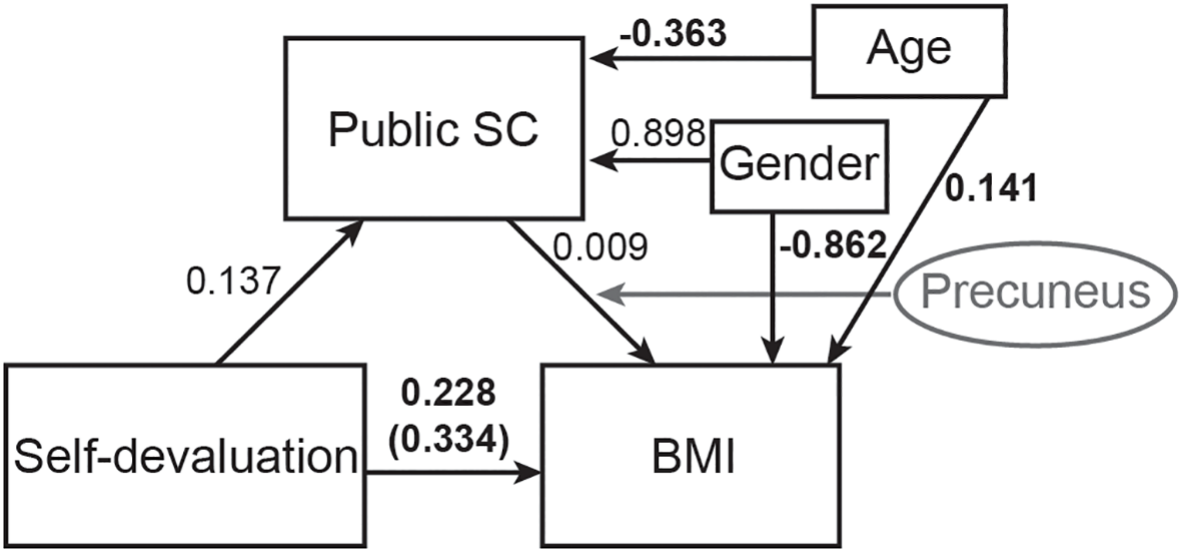
The diagram of the moderated mediation analysis. Gray matter volume in the precuneus was set as the moderator, and self-devaluation was set as the mediator. The standardized regression coefficients for the relationships are presented on the paths. The standardized regression coefficient between self-devaluation and BMI (direct effect), controlling for gray matter volume in the precuneus, public self-consciousness, gender, and age, is provided in parentheses. The gray line indicate moderation by gray matter volume in the precuneus on the path. Values in bold indicate significance, p < 0.05. BMI: body mass index, SC: self-consciousness.

Since the fMRI connectivity analysis showed an association between public self-consciousness and sACC-precuneus connectivity and the VBM analysis showed a positive association between public self-consciousness and precuneus gray matter volumes, gray matter volumes in the precuneus would moderate the path from public self-consciousness to BMI. To test this hypothesis, the moderated mediation analysis was conducted (**Figure 5**). The Index of Moderated Mediation (IMM), which is the rate of change of the indirect effect per 1-unit increase in the moderator, was 0.072, with a 95% CI [−0.134, 0.398], indicating no reliable evidence that the indirect effect changes with gray matter volumes in the precuneus. The conditional indirect effect of self-devaluation on BMI via public self-consciousness was 0.006 (95% CI [−0.013, 0.032]) when gray matter volumes in the precuneus was +1 SD, 0.001 (95% CI [−0.011, 0.012]) at the mean, and −0.004 (95% CI [−0.032, 0.011]) at −1 SD. In each case, the 95% confidence interval included zero, indicating the indirect effect was not statistically significant at any probed moderator level. The a-path (X→M) was 0.137, indicating higher self-devaluation was associated with higher public self-consciousness. The simple slope of M→Y varied with gray matter volumes in the precuneus: 0.047 at gray matter volumes in the precuneus = +1 SD, 0.009 at gray matter volumes in the precuneus = mean (0), and −0.030 at gray matter volumes in the precuneus = −1 SD. A Wald test on the interaction term (M×W) was χ² (1) = 2.97, p = 0.085, indicating the moderation of the M→Y path was not statistically significant at α = .05.

## 4 Discussion

The current study examined the associations among WBI, self-consciousness, and BMI in Japan, South Korea, Germany, and the United States. Additionally, the study explored the associations between WBI or self-consciousness, and the brain’s structural and functional properties.

As indicated in a previous study (9), self-devaluation demonstrated a positive association with BMI in Japan and the other countries, among both men and women, except for men in Germany and the United States. WBI was significantly associated with dysregulated eating behaviors, such as overeating (8), that were used to cope with WBI’s negative psychological effects (22), which could potentially cause weight gain (8) or weight regain (11). Contrary to the hypothesis, public self-consciousness was negatively associated with BMI in Japan, South Korea, and the United States. A significant preference for thinness exists in Asian and Western countries (52), and there is a common belief that weight can be controlled through diet and exercise (7), and that individuals who lack the willpower to control these habits are significantly susceptible to weight gain (22). Thus, given that public self-consciousness is the awareness of the self as a social and public object (18), individuals with greater public self-consciousness may be more sensitive to the pressure to remain thin for both aesthetic and social reasons. Furthermore, since public self-consciousness denotes attentiveness to the self as viewed by others (18), it would help individuals recognize themselves objectively. Thus, in the same way that keeping a daily food diary helps to objectively recognize one’s own eating habits, change eating behavior, and lead to significant weight loss (53), public self-consciousness would help to objectively monitor individuals’ eating habits and maintain healthy diet. Additionally, as the results of regression analysis and model comparison analysis revealed, self-devaluation could be a predictor of weight gain, whereas public self-consciousness demonstrated a mediation effect, mitigating the weight gain driven by self-devaluation in the Japanese. Overall, self-devaluation could be a predictor rather than an outcome of weight gain, whereas public self-consciousness may attenuate the positive impact of self-devaluation on weight gain.

Unlike in previous studies, fear of enacted stigma, but not self-devaluation, was not significantly associated with BMI in samples from all countries. We do not have a definitive explanation for this discrepancy. The current study used multiple linear regression analysis to test this association, whereas previous studies used bivariate correlation analysis (9, 38). These differences in statistical methods could explain the discrepancy. One possibility is that self-devaluation is correlated with psychological distress, such as anxiety, depression, and stress, better than fear of enacted stigma (38). Since greater psychological distress is associated with higher body weight (54), self-devaluation, rather than fear of enacted stigma, was significantly associated with BMI.

Since the majority of previous studies on WBI have been conducted in the United States with women with excess weight (2), conducting WBI studies in other regions with populations that have a wider range of BMI seemed crucial. Thus, the present study was conducted in four countries among individuals with wide-ranging BMI. The mediation effect of public self-consciousness on the relationship between self-devaluation and BMI differed across Japan, South Korea, Germany, and the United States, although the difference was not statistically significant. Previous studies reveal cultural differences in public self-consciousness (23). For example, power distance—the distance a person feels or maintains between themselves and a person in a position of power (55), which is pronounced in Asian countries (56)—positively affected public self-consciousness, which in turn positively influenced consumers’ intention to eat healthfully (25). Moreover, because our data revealed significant mediating effects of public self-consciousness among women from Japan, a gender effect on cultural differences in the mediating effect of public self-consciousness is plausible. Public self-consciousness had a more substantial influence on the internalization of ideal appearance among South Korean females than among German females (24). Thus, Asian women may be more affected by public self-consciousness, which can lead to an internalization of a preference for thinness. Consequently, the positive effect of self-devaluation on BMI may be mitigated. In fact, from 1990 to 2022, although the prevalence of underweight people declined in the majority of the 200 countries for both men and women, South Korea and Japan were the sole regions to exhibit an epidemiologically significant increase in prevalence of being underweight among women, notwithstanding their classification as high-income nations (1). Additionally, in comparison to the other three studied countries, self-devaluation exhibited a stronger positive correlation with BMI in Japan, despite Japan having the lowest BMI among them. Thus, public self-consciousness likely exerts a more pronounced negative mediating effect on the relationship between self-devaluation and BMI in Japanese women. Consequently, the negative mediating effect of public self-consciousness on BMI may vary across countries and is particularly pronounced among women in Japan although there are cultural and regional variations in factors influencing weight maintenance, such as genetic, biological, and environmental elements.

Structural brain imaging data showed that public self-consciousness was positively associated with gray matter volume in the lateral occipital cortex and precuneus, though the association was marginally significant, and these results are consistent with a previous MRI study (57). The precuneus and its surrounding regions, including the PCC, could play significant roles in self-awareness, mental representations related to oneself (58), and speculating or understanding how others perceive one’s physical and personality traits (e.g., “I think my friend thinks I am selfish”) (59). In addition, because precuneus activity during rest is sensitive to the extent to which an individual’s self-esteem is influenced by others’ evaluations, the precuneus is involved in integrating subjective interpretations of these evaluations with self-esteem (60). Consequently, the precuneus and the lateral occipital cortex may play a role in self-recognition by considering how others judge an individual.

Subsequently, the sACC response was positively related to self-devaluation, and greater sACC–PCC/precuneus connectivity was positively related to public self-consciousness. The sACC has a wide range of neural connections with various regions including the precuneus (26), and is involved with food-reward processing (28), social prediction errors (30, 31), and observation-driven social learning (32). Social approval prediction errors, which were calculated as the difference between the feedback received from others (e.g., how likable the person was) and the participants’ expected social feedback, were significantly associated with the sACC (31). Such prediction errors in the sACC could be used for observation-driven social learning through direct experience or by observing the action and outcome of another person (32). Thus, the sACC could play a role in internalizing negative stereotypes about people with higher weights. The sACC is also involved in food reward processing (28). Furthermore, people who have experienced weight-based discrimination are more likely to eat for the rewarding and relieving aspects of highly palatable food (61). Thus, weight bias internalization would influence food reward-driven over-eating. Therefore, during palatable liquid consumption, the sACC might evaluate food reward under the influence of the degree of WBI. Furthermore, greater sACC–precuneus/PCC connectivity was positively associated with public self-consciousness. Alterations in sACC and precuneus/PCC connection have been linked to eating disorders (62) and depression (63), which is characterized by a lack of motivation and anhedonia. Thus, this neural connection could play a role in processing food-reward value. Overall, public self-consciousness exhibited a negative mediating effect on the positive impact of self-devaluation on BMI, and greater public self-consciousness was associated with increased gray matter volumes in the precuneus and greater sACC–precuneus/PCC functional connectivity, suggesting that public self-consciousness may have a suppressing mediating effect on the positive associations between self-devaluation and BMI, and this effect of public self-consciousness would be associated with the precuneus/PCC.

This study has some limitations. First, although the online survey suggests that self-devaluation is a predictor rather than a consequence of weight gain, the causal relationship between weight gain and self-devaluation should be confirmed by longitudinal studies. Second, this study focused on WBI, BMI, and self-consciousness, ignoring factors like socioeconomic status (6), which significantly influence WBI. Future studies should consider other factors to better understand WBI. Third, public self-consciousness was linked to gray matter volume in the precuneus, but this region doesn’t overlap with the precuneus/PCC cluster seen in the connectivity analysis. Since the precuneus may exhibit enhanced connectivity with nearby areas as well as intra-hemispheric connectivity (64), the current findings would indicate that public self-consciousness is controlled across the precuneus and adjacent regions. However, it is important to note that differences in participant or brain imaging techniques between structural and functional brain imaging could potentially account for the observed discrepancies in brain imaging findings. Fourth, brain measures were performed on Japanese samples only. Cultural differences found among WBI, BMI, and public self-consciousness make a cross-cultural brain imaging comparison essential for future studies. Finally, the sample size for the fMRI experiment would be relatively small. However, a previous systematic review of fMRI studies using gustatory stimuli revealed that the median sample size was 24 participants (65). Additionally, the standardized coefficients of the linear regression analysis showing the link between brain response and self-devaluation were 0.72. This indicates a large effect, as effect sizes of 0.10–0.29 are small, 0.30–0.49 are medium, and 0.50 or greater are large (66). Thus, the sample size could be acceptable. However, future studies should have a greater sample size.

The current study has found that public self-consciousness would negatively mediate the positive effect of self-devaluation on BMI, and its mediating effect varied across different cultures. Furthermore, brain measurements have shown that self-devaluation was associated with the sACC, while public self-consciousness was associated with the precuneus/PCC and sACC-PCC connectivity. Therefore, public self-consciousness may be related to self-devaluation via sACC-precuneus/PCC connectivity. Although excessive public self-consciousness would be associated with maladaptive eating (21), proper adjustment of public self-consciousness would be a potential therapeutic target to mitigate the negative health consequences of WBI.

## Data Availability

The original contributions presented in the study are included in the article/**Supplementary Material**. Further inquiries can be directed to the corresponding author.
