# Supplementary material for: Self-consciousness negatively mediates the positive association between internalized weight bias and weight status in cross-cultural survey and brain imaging study
Source: Front Psychiatry. 2025 Nov 3;16:1703291. doi: 10.3389/fpsyt.2025.1703291 (PMC12620435; doi:10.3389/fpsyt.2025.1703291)
Supplement: Supplementary file 1 [file DataSheet1.docx]

Supplementary Material

# Supplementary Methods

## Participants

### Online survey

All online surveys were performed by commercial survey sampling and administration companies. From Japan, a total of 1972 participants completed the online survey between June 11, 2022 and June 13, 2022, and between February 16, 2024 and February 29, 2024. In total, 26 participants were excluded from analysis due to incomplete weight, height, or age data. From South Korea, a total of 1946 participants were included in the statistics. A total of 500 participants completed the online survey between November 20, 2024 and November 21, 2024. No participants failed to enter their responses, thus all 500 participants were included in the statistics. From Germany, a total of 598 participants completed the online survey between November 21, 2024 and November 25, 2024. No participants failed to enter their responses, resulting in a total of 598 participants included in the statistics. From the United States, a total of 582 participants completed the online survey between November 21, 2024 and November 25, 2024. Two participants were excluded from the analysis due to incomplete weight or height data. A total of 580 participants were included in the statistics.

### Differences in demographics in studied countries

Age, body mass index (BMI), and male-to-female ratio were compared in four countries: Japan, South Korea, Germany, and the United States. The Kruskal–Wallis rank sum test indicated that age and BMI differed significantly among the four countries (age:χ^2^(3)= 181.21, p < 0.001, BMI:χ^2^(3)= 575.99, p < 0.001). The post-hoc test revealed that age in Japan was the highest (ps_Bonferroni-corrected_ < 0.001), while age in Germany was the lowest (ps_Bonferroni-corrected_ < 0.001). BMI in Japan was the lowest (ps_Bonferroni-corrected_ < 0.001), and BMI in the United States was the highest across the four countries (ps_Bonferroni-corrected_ < 0.001). A Chi-squared test indicated that male-to-female ratio differed significantly across four countries (χ^2^(3)= 63.76, p < 0.001). The Kruskal–Wallis rank sum test indicated that age and BMI differed significantly among the four countries (age:χ^2^(3)= 87.62, p < 0.001, BMI:χ^2^(3)= 147.95, p < 0.001) in men and (age:χ^2^(3)= 124.66, p < 0.001, BMI:χ^2^(3)= 404.80, p < 0.001) in women. In men, the post-hoc test indicated that age was highest in Japan (ps_Bonferroni-corrected_ < 0.01), whereas age was lowest in Germany (ps_Bonferroni-corrected_ < 0.013). BMI in Japan was the lowest (ps_Bonferroni-corrected_ < 0.001), and BMI in the United States was the highest among the four countries (ps_Bonferroni-corrected_ < 0.05). Among women, the post-hoc test showed that age was higher in Japan than in South Korea and Germany (ps_Bonferroni-corrected_ < 0.006), but there was no significant difference in age between Japan and the United States. Age in Germany was the lowest among the four countries (ps_Bonferroni-corrected_ < 0.023). The post-hoc test also showed that BMI was lowest in Japan (ps_Bonferroni-corrected_ < 0.005) and highest in the United States among the four countries (ps_Bonferroni-corrected_ < 0.001).

## Measurements for the online survey

### The weight self-stigma questionnaire (WSSQ)

To assess weight bias internalization (WBI), the WSSQ (1) was used (Table S1). This is a 12-item self-report measure comprising two subscales: self-devaluation and fear of enacted stigma. Responses are rated on a 5-point Likert scale, ranging from 1 (strongly disagree) to 5 (strongly agree), with higher scores indicating greater levels of WBI. The WSSQ translated into the national language of each country was utilized for participants from Japan (2), South Korea (3), and Germany (4).

### The Self-Consciousness Scale (SCS)

To assess public- and private self-consciousness, the SCS (5) was used (Table S2). For all participants from the United States, the original version of the SCS was adapted. It contains 7 and 10 items evaluating public and private self-consciousness, respectively. Each item is rated on a 5-point Likert scale ranging from 1 (extremely uncharacteristic) to 5 (extremely characteristic). Korean and German versions of the SCS were used for all participants from South Korea and Germany, respectively. The Korean (6) and German (7) versions of the SCS were translated from the original SCS, and their reliability and validity were confirmed. For all participants from Japan, the Japanese version of the SCS was used (5). The Japanese version of the SCS was developed based on the original SCS (8), and its reliability and validity was confirmed. The Japanese version of the SCS consists of 11 and 10 items evaluating public and private self-consciousness, respectively. Each of the 21 items is rated on a 7-point Likert scale ranging from 1 (strongly disagree) to 7 (strongly agree).

### The reliability of the Weight Self-Stigma Questionnaire and the Self-Consciousness Scale

The reliability (internal consistency) of the SCS and WSSQ was estimated by calculating Cronbach’s α in each dataset. Cronbach's alpha coefficients for the public SCS, private SCS, self-devaluation, and fear of enacted stigma were greater than 0.70 in the Japanese, South Korean, German, and American samples, except for the private SCS in the German (0.69) and American (0.68) samples. Given that Cronbach’s alpha of 0.70 and above is acceptable (9), the internal consistency of the WSSQ and public SCS was determined to be good although private SCS in the German and American samples may be questionable.

Among men, Cronbach's alpha coefficients for the public SCS, private SCS, self-devaluation, and fear of enacted stigma were greater than 0.70 in the Japanese, South Korean, German, and American samples, except for the private SCS in the German sample (0.68). For women, Cronbach's alpha coefficients for the public SCS, private SCS, self-devaluation, and fear of enacted stigma were greater than 0.70 in the Japanese, South Korean, German, and American samples, except for the private SCS in the German (0.69) and American (0.64) samples.

## Statistics

### Online survey

#### Multiple linear regression analysis

The R statistical software (v4.4.1; R Foundation for Statistical Computing, Vienna, Austria) was used for this analysis. To scale each variable, all numeric variables were centered and divided by the standard deviation. In order to assess multicollinearity of the regression model, the variance inflation factor (VIF) was calculated for each of the predictor variables. The resulting VIF values were all less than 1.97. Given that VIF values greater than 5 indicate problematic amounts of collinearity (10), it was assumed that there was no serious multicollinearity problem with this model. Then, the same regression model was developed using the same variables used in all participants, except for gender, in men and women separately. The VIFs were less than 1.84 in men and 2.02 in women. Thus, it was assumed that there was no serious multicollinearity problem with the models.

For data in the samples from South Korea, Germany, and the United States, the same regression models were used as that used for the sample from Japan. For this analysis, scaled numeric variables were used. VIFs from all samples were less than 3.16, and it was assumed that there was no serious multicollinearity problem with the models.

### Brain imaging data

#### Structural images

Initially, the apparent artifacts and image quality were inspected visually, which confirmed the high quality of the images. Then, the CAT12.9 (r2577) (https://neuro-jena.github.io/cat/) was used to assess the quality of images. The CAT 12 adapts a retrospective quality control framework for empirical quantification of quality differences in different scans or studies. This framework allows the evaluation of essential image parameters such as noise, inhomogeneities, and image resolution. All these quality measures are combined into a single quality score known as image quality rating (IQR). The IQR is a weighted average of the local (noise contrast ratio) and global (inhomogeneity contrast ratio) SDs within the optimized white matter segment, scaled by the minimum tissue contrast and the root mean square of the voxel size. The obtained IQRs range from 0.5 to 10.5, with values around 1 and 2 describing excellent or good image quality (Grades A and B, respectively) and values 5 (Grade E) and higher (Grade F) indicating problematic images. The IQRs from this study were 1.847 ± 0.004 (mean ± SD), ranging from 1.834 to 1.868, indicating that all anatomical images had good-to-excellent image quality.

For brain morphometry analyses of T1-weighted structural images, voxel-based morphometry (VBM) was applied. All structural images were analyzed with FSL-VBM (11), an optimized VBM protocol (12) implanted in FSL v6.0.7.15 (13). First, structural images were skull-stripped and grey matter-segmented prior to being registered to the Montreal Neurological Institute (MNI) 152 standard space via non-linear registration. The resulting images were averaged and flipped along the x-axis to create a left-right symmetric, study-specific grey matter template. Second, all native grey matter images were non-linearly registered to the study-specific template and "modulated" to correct for local expansion (or contraction) due to the non-linear component of the spatial transformation. The modulated grey matter images were then smoothed with an isotropic Gaussian kernel with a sigma of 3 mm. Finally, a voxel-wise general linear model (GLM) was applied using permutation-based non-parametric testing with 5,000 permutations (14). To examine the association between gray matter volumes and weight self-stigma or self-consciousness, the GLM included preprocessed grey matter images as a dependent variable, WSSQ or SCS as explanatory variables, and BMI and estimated total intracranial volume (eTIV) as nuisance variables. eTIV was calculated with FreeSurfer v7.4.1 (https://surfer.nmr.mgh.harvard.edu). The predicted effect of these analyses was tested using a region of interest (ROI) approach. Threshold-free cluster enhancement (TFCE) was employed to assess cluster significance (15), with threshold of p_family-wise error rate (FWE)-corrected_ < 0.05.

#### Functional images

All functional and anatomical data underwent preprocessing using Statistical Parametric Mapping 12 (SPM12)(16) software with the CONN functional connectivity toolbox (CONN, version 22a)(17, 18). Functional and anatomical data were preprocessed using a modular preprocessing pipeline (19) including slice timing correction, creation of voxel-displacement maps, realignment with susceptibility distortion correction using fieldmaps, outlier detection, indirect segmentation and MNI-space normalization, and smoothing. Temporal misalignment between different slices of the functional data (acquired in interleaved bottom-up order) was corrected following SPM slice-timing correction (STC) procedure (20, 21), using sinc temporal interpolation to resample each slice BOLD timeseries to a common mid-acquisition time. Functional data were realigned using SPM realign & unwarp procedure (22) integrating fieldmaps for susceptibility distortion correction, where all scans were coregistered to a reference image (first scan of the first session) using a least squares approach and a 6 parameter (rigid body) transformation, and resampled using b-spline interpolation (23) to simultaneously correct for motion, magnetic susceptibility geometric distortions, and their interaction. Potential outlier scans were identified using ART (24) as acquisitions with framewise displacement above 0.9 mm or global BOLD signal changes above 5 standard deviations (25), and a reference BOLD image was computed for each subject by averaging all scans, excluding outliers. Functional and anatomical data were coregistered and normalized into standard MNI space, segmented into grey matter, white matter, and CSF tissue classes, and resampled to 2 mm isotropic voxels following an indirect normalization procedure (26) using SPM unified segmentation and normalization algorithm (27, 28) with the default IXI-549 tissue probability map template. Last, functional data were smoothed using spatial convolution with a Gaussian kernel of 6 mm full width half maximum (FWHM).

# Supplementary Figures and Tables

## Supplementary Figures


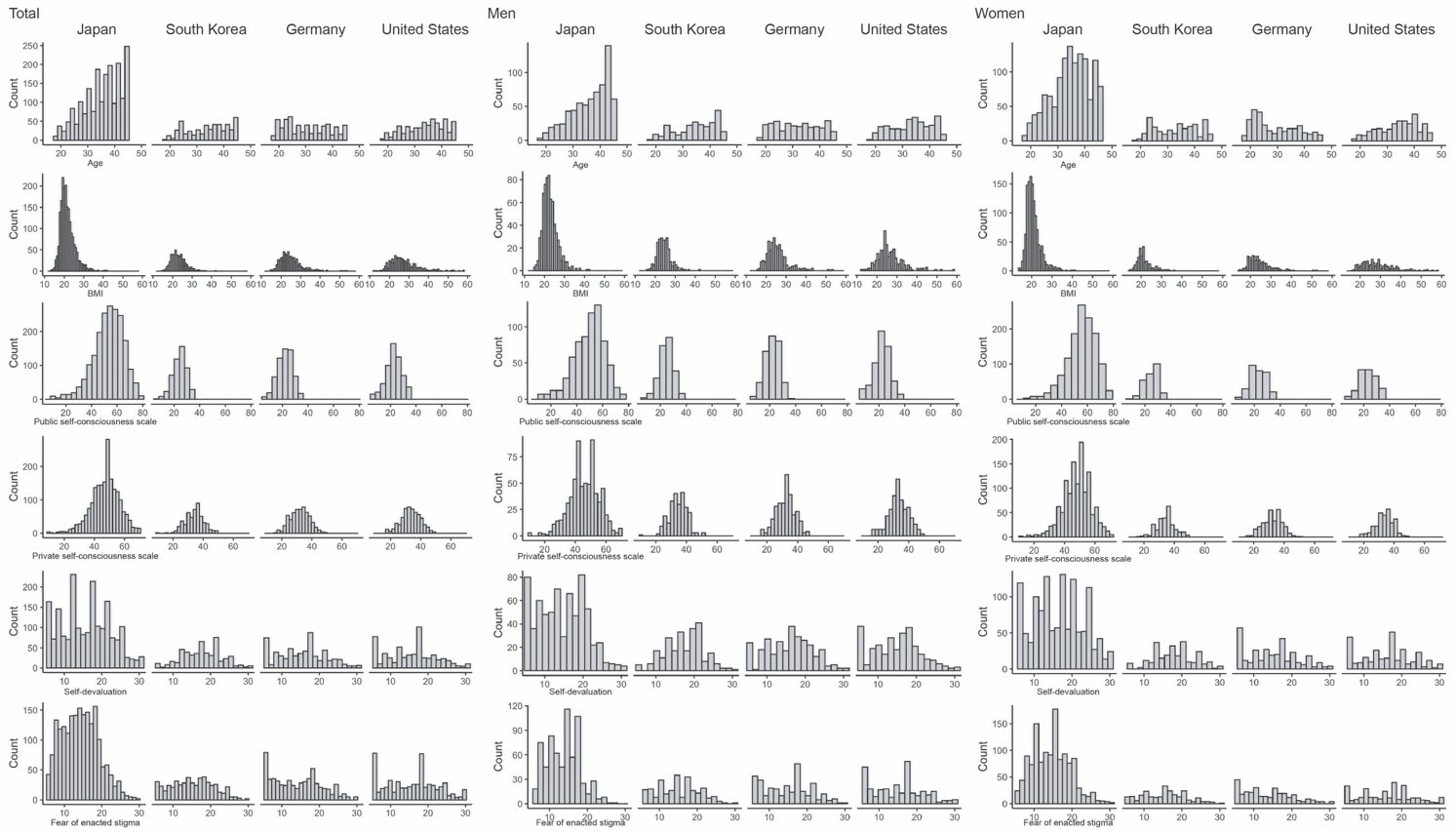
**Supplementary Figure 1.** Histograms of age, BMI, SCS, and WSSQ. BMI: body mass index, SCS: self-conscious scale, WSSQ: weight self-stigma questionnaire.

**
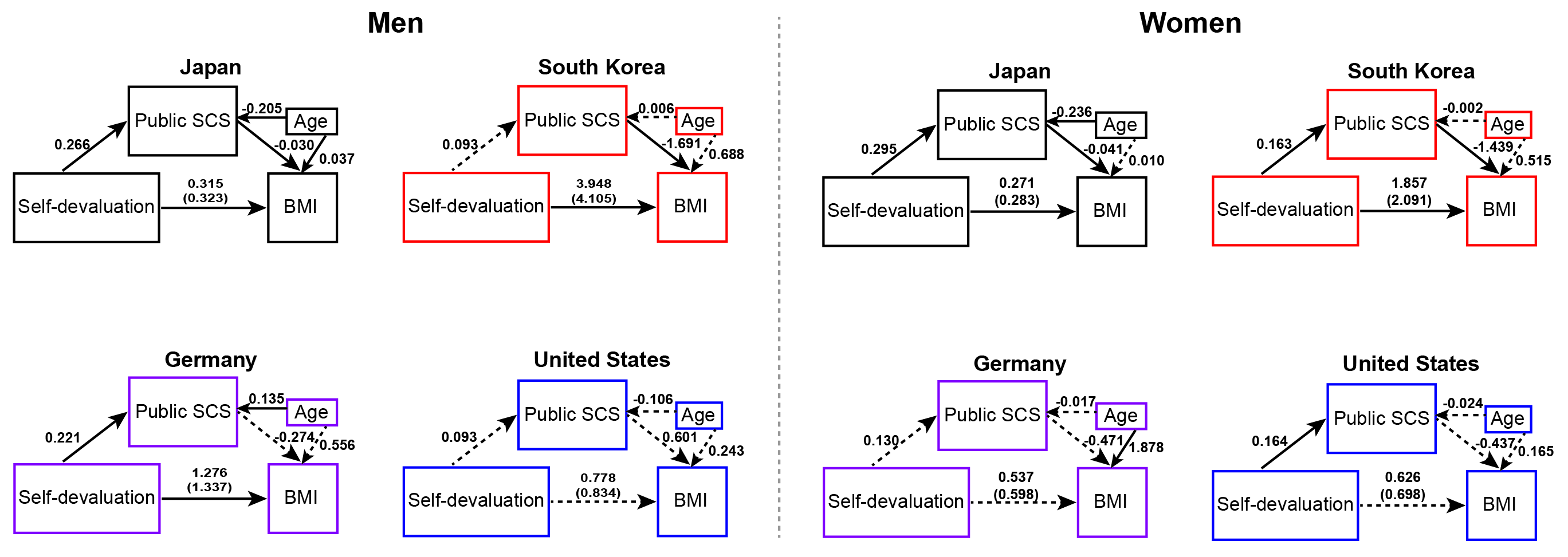
Supplementary Figure 2.** Associations among self-devaluation of the Weight Self-Stigma Questionnaire, public self-consciousness of the Self-Consciousness Scale (public SCS), and body mass index (BMI) in men and women of the Japanese, South Korean, German, and the American samples. The standardized regression coefficients for the relationships are presented on the paths. The standardized regression coefficient between self-devaluation and BMI (direct effect), controlling for public self-consciousness, is provided in parentheses. Dashed line paths indicate non-significance, p = 0.05 or more. Solid line paths indicate significance, p < 0.05. BMI: body mass index; Public SCS: public self-consciousness of the self-conscious scale.


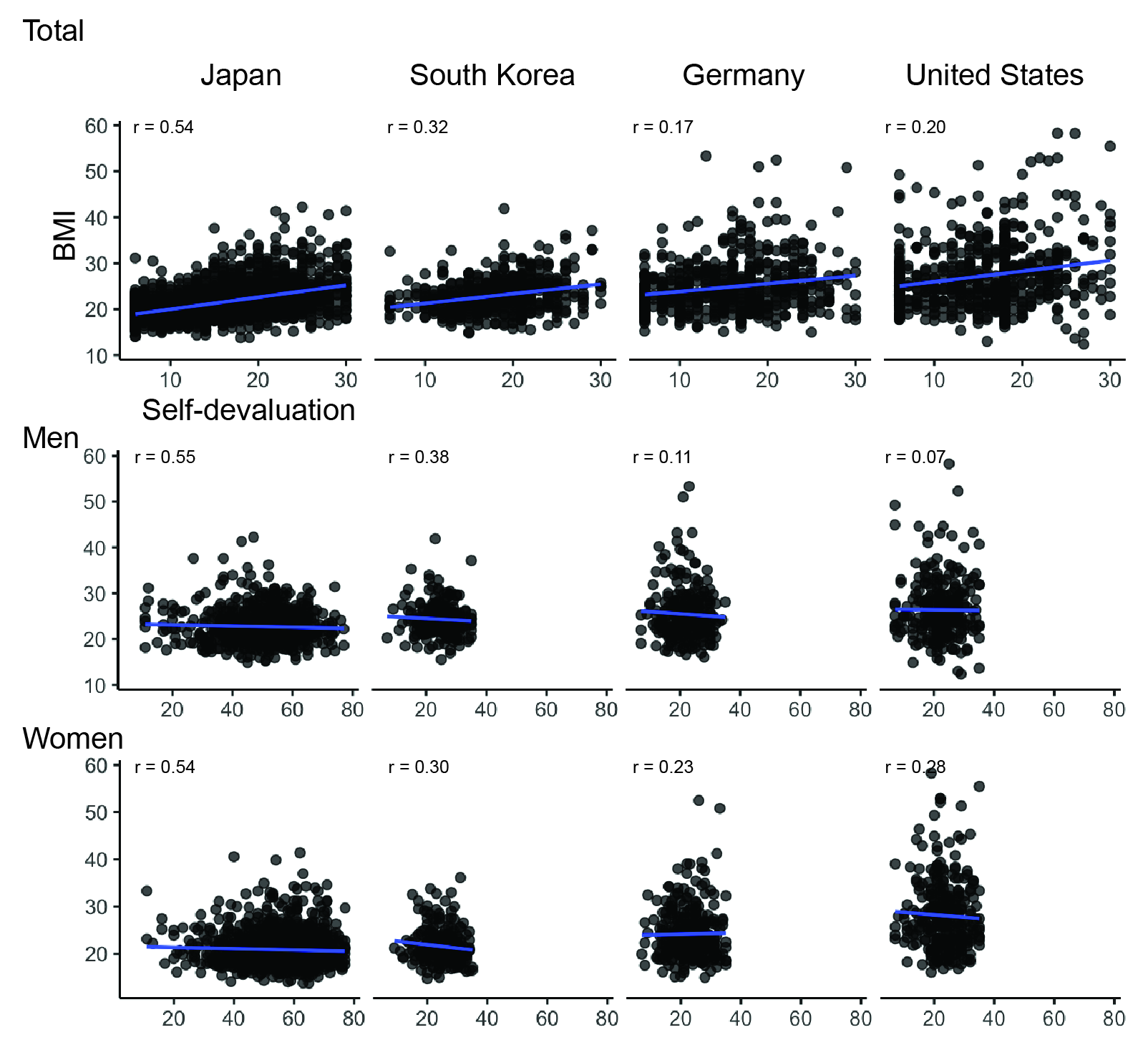


**Supplementary Figure 3.** Scatter plots representing BMI and self-devaluation. Blue lines depict the simple regression lines. BMI: body mass index. r: Spearman’s rhos, after controlling for age and gender.

## Supplementary Tables

**Table S1.** The associations between demographics and the Weight Self-Stigma Questionnaire and the Self-Consciousness Scale

|  | Age | BMI | Self-devaluation | Fear of enacted stigma | Public self-consciousness | Private self-consciousness |
| --- | --- | --- | --- | --- | --- | --- |
| Japan (men and women) | | | | | | |
| Age | 1.00 | **0.13**  (< 0.01) | **0.05**  (0.03) | 0.00  (0.96) | **-0.18**  (< 0.01) | **-0.14**  (< 0.01) |
| BMI | - | 1.00 | **0.47**  (< 0.01) | **0.29**  (< 0.01) | **-0.10**  (< 0.01) | **-0.05**  (0.02) |
| South Korea (men and women) | | | | | | |
| Age | 1.00 | **0.22**  (< 0.01) | **0.17**  (< 0.01) | 0.04  (0.37) | **-0.12**  (0.01) | **-0.12**  (0.01) |
| BMI | - | 1.00 | **0.28**  (< 0.01) | **0.16**  (< 0.01) | **-0.10**  (0.03) | -0.02  (0.58) |
| Germany (men and women) | | | | | | |
| Age | 1.00 | **0.26**  (< 0.01) | **0.08**  (0.04) | **-0.08**  (0.05) | 0.02  (0.60) | 0.06  (0.17) |
| BMI | - | 1.00 | **0.20**  (< 0.01) | 0.05  (0.19) | -0.04  (0.38) | -0.04  (0.33) |
| United States (men and women) | | | | | | |
| Age | 1.00 | **0.21**  (< 0.01) | 0.01  (0.73) | -0.05  (0.19) | **-0.11**  (0.01) | **-0.10**  (0.02) |
| BMI | - | 1.00 | **0.21**  (< 0.01) | **0.12**  (< 0.01) | -0.02  (0.63) | 0.04  (0.28) |
| Japan (men) | | | | | | |
| Age | 1.00 | **0.17**  (< 0.01) | **0.15**  (< 0.01) | 0.05  (0.21) | **-0.13**  (< 0.01) | **-0.14**  (< 0.01) |
| BMI | - | 1.00 | **0.56**  (< 0.01) | **0.30**  (< 0.01) | -0.01  (0.83) | 0.00  (0.96) |
| South Korea (men) | | | | | | |
| Age | 1.00 | **0.14**  (0.03) | **0.13**  (0.04) | 0.05  (0.44) | -0.10  (0.13) | -0.10  (0.12) |
| BMI | - | 1.00 | **0.39**  (< 0.01) | **0.25**  (< 0.01) | -0.07  (0.28) | -0.05  (0.41) |
| Germany (men) | | | | | | |
| Age | 1.00 | **0.30**  (< 0.01) | 0.06  (0.32) | **-0.15**  (0.01) | **0.14**  (0.02) | **0.13**  (0.03) |
| BMI | - | 1.00 | **0.12**  (0.04) | -0.02  (0.72) | -0.02  (0.68) | -0.03  (0.64) |
| United States (men) | | | | | | |
| Age | 1.00 | **0.21**  (< 0.01) | 0.02  (0.71) | -0.07  (0.24) | -0.06  (0.29) | -0.04  (0.45) |
| BMI | - | 1.00 | 0.07  (0.24) | 0.00  (0.98) | 0.02  (0.75) | **0.17**  (< 0.01) |
| Japan (women) | | | | | | |
| Age | 1.00 | **0.06**  (0.04) | 0.02  (0.41) | -0.01  (0.62) | **-0.17**  (< 0.01) | **-0.12**  (< 0.01) |
| BMI | - | 1.00 | **0.54**  (< 0.01) | **0.33**  (< 0.01) | -0.04  (0.15) | -0.02  (0.39) |
| South Korea (women) | | | | | | |
| Age | 1.00 | **0.22**  (< 0.01) | **0.22**  (< 0.01) | 0.04  (0.55) | **-0.14**  (0.03) | **-0.15**  (0.02) |
| BMI | - | 1.00 | **0.34**  (< 0.01) | **0.15**  (0.01) | -0.10  (0.13) | -0.06  (0.34) |
| Germany (women) | | | | | | |
| Age | 1.00 | **0.18**  (< 0.01) | 0.08  (0.16) | -0.04  (0.43) | -0.06  (0.33) | -0.01  (0.84) |
| BMI | - | 1.00 | **0.24**  (< 0.01) | 0.09  (0.12) | -0.03  (0.61) | -0.06  (0.32) |
| United States (women) | | | | | | |
| Age | 1.00 | **0.19**  (< 0.01) | -0.01  (0.86) | -0.05  (0.35) | **-0.17**  (< 0.01) | **-0.16**  (0.01) |
| BMI | - | 1.00 | **0.28**  (< 0.01) | **0.21**  (< 0.01) | -0.06  (0.28) | -0.07  (0.23) |

Spearman’s rhos are presented. P-values are provided in parentheses. BMI: body mass index

**Table S2.** The weight self-stigma questionnaire

| Instructions: Please select one of the following five options to indicate how well each item applies to you:  1. Completely disagree, 2. Disagree, 3. Neither disagree nor agree, 4. Agree, 5. Completely agree. |
| --- |
| 1. I’ll always go back to being overweight. |
| 2. I caused my weight problems. |
| 3. I feel guilty because of my weight problems. |
| 4. I became overweight because I’m a weak person. |
| 5. I would never have any problems with weight if I were stronger. |
| 6. I don’t have enough self-control to maintain a healthy weight. |
| 7. I feel insecure about others’ opinions of me. |
| 8. People discriminate against me because I’ve had weight problems. |
| 9. It’s difficult for people who haven’t had weight problems to relate to me. |
| 10. Others will think I lack self-control because of my weight problems. |
| 11. People think that I am to blame for my weight problems. |
| 12. Others are ashamed to be around me because of my weight. |

Items 1 to 6 comprise the self-devaluation subscale and items 7 to 12 the fear of enacted stigma subscale.

**Table S3.** The self-conscious scale

| Instructions: Please select one of the following five options to indicate how well each item applies to you:  1. Extremely uncharacteristic, 2. Somewhat uncharacteristic, 3. Neither uncharacteristic nor characteristic, 4. Somewhat characteristic, 5. Extremely characteristic. |
| --- |
| 1. I'm always trying to figure myself out. |
| 2. I'm concerned about my style of doing things. |
| 3. Generally, I'm not very aware of myself. |
| 4. I reflect about myself a lot. |
| 5. I'm concerned about the way I present myself. |
| 6. I'm often the subject of my own fantasies. |
| 7. I never scrutinize myself. |
| 8. I'm self-conscious about the way I look. |
| 9. I'm generally attentive to my inner feelings. |
| 10. I usually worry about making a good impression. |
| 11. I'm constantly examining my motives. |
| 12. One of the last things I do before I leave my house is look in the mirror. |
| 13. I sometimes have the feeling that I'm off somewhere watching myself. |
| 14. I'm concerned about what other people think of me. |
| 15. I'm alert to changes in my mood. |
| 16. I'm usually aware of my appearance. |
| 17. I'm aware of the way my mind works when I work through a problem. |

Items 1, 3, 4, 6, 7, 9, 11, 13, 15, and 17 constitute the private self-consciousness subscale, and items 2, 5, 8, 10, 12, 14, and 16 constitute the public self-consciousness subscale. Items 3 and 7 are reversed for scoring.

**Table S4.** Ratings for internal state and the gustatory stimulus

|  | Hunger | Fullness | Liking | Wanting | Intensity | Familiarity |
| --- | --- | --- | --- | --- | --- | --- |
| Mean ± SD | 4.10 ± 1.79 | 4.23 ± 2.01 | 5.67 ± 1.30 | 5.17 ± 1.49 | 5.63 ± 1.30 | 5.47 ± 1.87 |
| Range | 1 - 8 | 1 - 7 | 3 - 8 | 2 - 7 | 2 - 8 | 1 - 8 |

**Table S5.** Results of the linear model fitting including BMI as the dependent variable

| Predictors | β | 95% confidence interval*  (lower, upper) | p-value |
| --- | --- | --- | --- |
| All participants |  |  |  |
| Japan |  |  |  |
| Self-devaluation | 0.53 | 0.49, 0.59 | **< 0.001** |
| Fear of enacted stigma | -0.02 | -0.08, 0.04 | 0.466 |
| Public self-consciousness | -0.12 | -0.16, -0.07 | **< 0.001** |
| Private self-consciousness | 0.00 | -0.04, 0.05 | 0.884 |
| Age | 0.04 | -0.00, 0.08 | **0.048** |
| Gender | -0.60 | -0.68, -0.53 | **< 0.001** |
| South Korea |  |  |  |
| Self-devaluation | 0.31 | 0.17, 0.42 | **< 0.001** |
| Fear of enacted stigma | 0.01 | -0.09, 0.12 | 0.841 |
| Public self-consciousness | -0.15 | -0.26, -0.04 | **0.008** |
| Private self-consciousness | 0.05 | -0.07, 0.15 | 0.396 |
| Age | 0.09 | 0.01, 0.18 | **0.037** |
| Gender | -0.75 | -0.90, -0.59 | **< 0.001** |
| Germany |  |  |  |
| Self-devaluation | 0.23 | 0.11, 0.35 | **< 0.001** |
| Fear of enacted stigma | -0.05 | -0.17, 0.10 | 0.491 |
| Public self-consciousness | -0.03 | -0.14, 0.12 | 0.710 |
| Private self-consciousness | -0.08 | -0.24, 0.03 | 0.236 |
| Age | 0.20 | 0.13, 0.28 | **< 0.001** |
| Gender | -0.13 | -0.29, 0.02 | 0.106 |
| United States |  |  |  |
| Self-devaluation | 0.23 | 0.11, 0.35 | **< 0.001** |
| Fear of enacted stigma | 0.01 | -0.11, 0.12 | 0.901 |
| Public self-consciousness | -0.18 | -0.28, -0.08 | **0.001** |
| Private self-consciousness | 0.14 | 0.03, 0.24 | **0.009** |
| Age | 0.14 | 0.06, 0.22 | **< 0.001** |
| Gender | 0.21 | 0.06, 0.37 | **0.011** |
| Men |  |  |  |
| Japan |  |  |  |
| Self-devaluation | 0.57 | 0.49, 0.66 | **< 0.001** |
| Fear of enacted stigma | -0.05 | -0.14, 0.04 | 0.240 |
| Public self-consciousness | -0.12 | -0.20, -0.04 | **0.002** |
| Private self-consciousness | 0.06 | -0.02, 0.14 | 0.144 |
| Age | 0.07 | 0.00, 0.13 | **0.032** |
| South Korea |  |  |  |
| Self-devaluation | 0.37 | 0.22, 0.55 | **< 0.001** |
| Fear of enacted stigma | 0.00 | -0.18, 0.15 | 0.952 |
| Public self-consciousness | -0.19 | -0.36, -0.04 | **0.023** |
| Private self-consciousness | 0.17 | 0.02, 0.35 | 0.053 |
| Age | 0.06 | -0.07, 0.18 | 0.352 |
| Germany |  |  |  |
| Self-devaluation | 0.15 | -0.02, 0.30 | 0.175 |
| Fear of enacted stigma | 0.00 | -0.16, 0.14 | 0.935 |
| Public self-consciousness | -0.13 | -0.29, 0.04 | 0.140 |
| Private self-consciousness | 0.00 | -0.14, 0.15 | 0.959 |
| Age | 0.26 | 0.09, 0.25 | **< 0.001** |
| United States |  |  |  |
| Self-devaluation | 0.21 | 0.01, 0.48 | 0.074 |
| Fear of enacted stigma | -0.08 | -0.29, 0.10 | 0.411 |
| Public self-consciousness | -0.21 | -0.38, -0.07 | **0.008** |
| Private self-consciousness | 0.25 | 0.10, 0.39 | **0.001** |
| Age | 0.13 | 0.02, 0.23 | **0.024** |
| Women |  |  |  |
| Japan |  |  |  |
| Self-devaluation | 0.53 | 0.46, 0.61 | **< 0.001** |
| Fear of enacted stigma | 0.00 | -0.08, 0.08 | 0.995 |
| Public self-consciousness | -0.12 | -0.18, -0.06 | **< 0.001** |
| Private self-consciousness | -0.03 | -0.08, 0.03 | 0.336 |
| Age | 0.02 | -0.03, 0.07 | 0.443 |
| South Korea |  |  |  |
| Self-devaluation | 0.29 | 0.09, 0.30 | **0.005** |
| Fear of enacted stigma | 0.03 | -0.08, 0.11 | 0.752 |
| Public self-consciousness | -0.14 | -0.21, 0.01 | 0.102 |
| Private self-consciousness | -0.04 | -0.12, 0.07 | 0.664 |
| Age | 0.13 | 0.00, 0.12 | **0.064** |
| Germany |  |  |  |
| Self-devaluation | 0.29 | 0.11, 0.37 | **< 0.001** |
| Fear of enacted stigma | -0.06 | -0.18, 0.08 | 0.482 |
| Public self-consciousness | 0.05 | -0.10, 0.19 | 0.621 |
| Private self-consciousness | -0.15 | -0.26, 0.00 | 0.160 |
| Age | 0.16 | 0.03, 0.18 | **0.005** |
| United States |  |  |  |
| Self-devaluation | 0.24 | 0.09, 0.38 | **0.003** |
| Fear of enacted stigma | 0.07 | -0.08, 0.22 | 0.379 |
| Public self-consciousness | -0.13 | -0.28, 0.02 | 0.094 |
| Private self-consciousness | 0.04 | -0.12, 0.19 | 0.645 |
| Age | 0.14 | 0.02, 0.25 | **0.013** |

* Bootstrap confidence intervals (1000 resamples)

**References**

1. Lillis J, Luoma JB, Levin ME, Hayes SC. Measuring Weight Self-Stigma: The Weight Self-Stigma Questionnaire. *Obesity (Silver Spring)* (2010) 18(5):971-6. Epub 20091015. doi: 10.1038/oby.2009.353.

2. Nakamura Y, Asano M. Developing and Validating a Japanese Version of the Weight Self-Stigma Questionnaire. *Eat Weight Disord* (2023) 28(1):44. Epub 20230517. doi: 10.1007/s40519-023-01573-0.

3. Park S, Seo K. Validity and Reliability of the Korean Version of the Weight Self-Stigma Questionnaire (Wssq-K). *Nurs Rep* (2023) 13(2):835-43. Epub 20230526. doi: 10.3390/nursrep13020073.

4. Hain B, Langer L, Hünnemeyer K, Rudofsky G, Zech U, Wild B. Translation and Validation of the German Version of the Weight Self-Stigma Questionnaire (Wssq). *Obes Surg* (2015) 25(4):750-3. doi: 10.1007/s11695-015-1598-6.

5. Fenigstein A, Scheier MF, Buss AH. Public and Private Self-Consciousness: Assessment and Theory. *Journal of Consulting and Clinical Psychology* (1975) 43(4):522-7. doi: 10.1037/h0076760.

6. Kang B, Shin H. Validation of the Self-Consciousness Scale for Korean Adolescents. *The Korean Journal of School Psychology* (2017) 14(1):105-28. doi: 10.16983/kjsp.2017.14.1.105.

7. Heinemann W. The Assessment of Private and Public Self-Consciousness: A German Replication. *European Journal of Social Psychology* (1979) 9(3):331-7. doi: 10.1002/ejsp.2420090311.

8. Sugawara K. An Attempting to Construct the Self-Consciousness Scale for Japanese. *The Japanese journal of psychology* (1984) 55(3):184-8. doi: 10.4992/jjpsy.55.184.

9. Cortina JM. What Is Coefficient Alpha? An Examination of Theory and Applications. *Journal of Applied Psychology* (1993) 78(1):98-104. doi: 10.1037/0021-9010.78.1.98.

10. James G, Witten D, Hastie T, Tibshirani R. *An Introduction to Statistical Learning: With Applications in R*: Springer New York (2013).

11. Douaud G, Smith S, Jenkinson M, Behrens T, Johansen-Berg H, Vickers J, et al. Anatomically Related Grey and White Matter Abnormalities in Adolescent-Onset Schizophrenia. *Brain* (2007) 130(Pt 9):2375-86. Epub 20070813. doi: 10.1093/brain/awm184.

12. Good CD, Johnsrude IS, Ashburner J, Henson RN, Friston KJ, Frackowiak RS. A Voxel-Based Morphometric Study of Ageing in 465 Normal Adult Human Brains. *Neuroimage* (2001) 14(1 Pt 1):21-36. doi: 10.1006/nimg.2001.0786.

13. Jenkinson M, Beckmann CF, Behrens TE, Woolrich MW, Smith SM. Fsl. *Neuroimage* (2012) 62(2):782-90. Epub 20110916. doi: 10.1016/j.neuroimage.2011.09.015.

14. Winkler AM, Ridgway GR, Webster MA, Smith SM, Nichols TE. Permutation Inference for the General Linear Model. *Neuroimage* (2014) 92(100):381-97. Epub 20140211. doi: 10.1016/j.neuroimage.2014.01.060.

15. Smith SM, Nichols TE. Threshold-Free Cluster Enhancement: Addressing Problems of Smoothing, Threshold Dependence and Localisation in Cluster Inference. *NeuroImage* (2009) 44(1):83-98. doi: 10.1016/j.neuroimage.2008.03.061.

16. Penny WD, Friston KJ, Ashburner JT, Kiebel SJ, Nichols TE. *Statistical Parametric Mapping: The Analysis of Functional Brain Images*: Academic Press (2011).

17. Nieto-Castanon A, Whitfield-Gabrieli S. *Conn Functional Connectivity Toolbox: Rrid Scr_009550, Release 22*: Hilbert Press (2022).

18. Whitfield-Gabrieli S, Nieto-Castanon A. Conn: A Functional Connectivity Toolbox for Correlated and Anticorrelated Brain Networks. *Brain Connect* (2012) 2(3):125-41. Epub 20120719. doi: 10.1089/brain.2012.0073.

19. Nieto-Castanon A. Fmri Minimal Preprocessing Pipeline. *Handbook of Functional Connectivity Magnetic Resonance Imaging Methods in Conn*. Hilbert Press (2020). p. 3-16.

20. Henson R, Buechel C, Josephs O, Friston KJ. The Slice-Timing Problem in Event-Related Fmri. *NeuroImage* (1999).

21. Sladky R, Friston KJ, Tröstl J, Cunnington R, Moser E, Windischberger C. Slice-Timing Effects and Their Correction in Functional Mri. *Neuroimage* (2011) 58(2):588-94. Epub 20110702. doi: 10.1016/j.neuroimage.2011.06.078.

22. Andersson JL, Hutton C, Ashburner J, Turner R, Friston K. Modeling Geometric Deformations in Epi Time Series. *Neuroimage* (2001) 13(5):903-19. doi: 10.1006/nimg.2001.0746.

23. Friston KJ, Ashburner J, Frith CD, Poline J-B, Heather JD, Frackowiak RSJ. Spatial Registration and Normalization of Images. *Human Brain Mapping* (1995) 3.

24. Whitfield-Gabrieli S, Nieto-Castanon A, Ghosh S. Artifact Detection Tools (Art). *Cambridge, MA Release Version* (2011) 7(19):11.

25. Power JD, Mitra A, Laumann TO, Snyder AZ, Schlaggar BL, Petersen SE. Methods to Detect, Characterize, and Remove Motion Artifact in Resting State Fmri. *Neuroimage* (2014) 84:320-41. Epub 20130829. doi: 10.1016/j.neuroimage.2013.08.048.

26. Calhoun VD, Wager TD, Krishnan A, Rosch KS, Seymour KE, Nebel MB, et al. The Impact of T1 Versus Epi Spatial Normalization Templates for Fmri Data Analyses. *Hum Brain Mapp* (2017) 38(11):5331-42. Epub 20170726. doi: 10.1002/hbm.23737.

27. Ashburner J, Friston KJ. Unified Segmentation. *Neuroimage* (2005) 26(3):839-51. Epub 20050401. doi: 10.1016/j.neuroimage.2005.02.018.

28. Ashburner J. A Fast Diffeomorphic Image Registration Algorithm. *Neuroimage* (2007) 38(1):95-113. Epub 20070718. doi: 10.1016/j.neuroimage.2007.07.007.

29. Flandin G, Friston KJ. Analysis of Family-Wise Error Rates in Statistical Parametric Mapping Using Random Field Theory. *Hum Brain Mapp* (2019) 40(7):2052-4. Epub 20171101. doi: 10.1002/hbm.23839.

30. Friston KJ, Buechel C, Fink GR, Morris J, Rolls E, Dolan RJ. Psychophysiological and Modulatory Interactions in Neuroimaging. *Neuroimage* (1997) 6(3):218-29. doi: 10.1006/nimg.1997.0291.

31. McLaren DG, Ries ML, Xu G, Johnson SC. A Generalized Form of Context-Dependent Psychophysiological Interactions (Gppi): A Comparison to Standard Approaches. *NeuroImage* (2012) 61:1277-86.

32. Worsley KJ, Marrett S, Neelin P, Vandal AC, Friston KJ, Evans AC. A Unified Statistical Approach for Determining Significant Signals in Images of Cerebral Activation. *Hum Brain Mapp* (1996) 4(1):58-73. doi: 10.1002/(sici)1097-0193(1996)4:1<58::Aid-hbm4>3.0.Co;2-o.
